# Comparative Genomics and Gene Cluster Family Analysis Reveal Conserved and Isolate-Specific Biosynthetic Potential in Medicinal Plant-Associated Endophytic *Diaporthe*

**DOI:** 10.3390/jof12100784

**Published:** 2026-10-20

**Authors:** Biju Vadakkemukadiyil Chellappan, Ashraf Abbass Metwally

**Affiliations:** Department of Biological Sciences, College of Science, King Faisal University, Al-Ahsa 31982, Saudi Arabia

**Keywords:** biosynthetic gene clusters, comparative genomics, *Diaporthe*, endophytic fungi, gene cluster families, medicinal plants

## Abstract

Endophytic fungi associated with medicinal plants represent promising reservoirs of specialized metabolites; however, the genomic diversity underlying their biosynthetic potential remains incompletely characterized. Here, we performed comparative genomic and genome-mining analyses of four medicinal plant-associated endophytic *Diaporthe* isolates: *D. inconspicua* LGMF1612, *D.* cf. *heveae* LGMF1633, *D. vochysiae* LGMF1583, and *Diaporthe* sp. HANT25. Genome sizes ranged from 52.45 to 60.11 Mb, with 16,033–17,434 predicted proteins. Comparative orthology identified 16,196 protein clusters, of which 11,230 (69.3%) were conserved across all four isolates, indicating a substantial shared proteomic repertoire accompanied by smaller variable components. Functional annotation revealed extensive representation of membrane transporters, fungal transcriptional regulators, cytochrome P450s, redox and detoxification enzymes, and protein domains associated with specialized metabolism. Genome mining using antiSMASH identified 481 biosynthetic gene clusters (BGCs), with 112–129 BGCs per isolate. Type I polyketide synthase clusters were predominant, together with substantial NRPS, NRPS-like, terpene, and hybrid/multiclass repertoires. BiG-SLiCE grouped the predicted BGCs into 348 gene cluster families (GCFs), only 15 of which were classified as core GCFs, with members represented in all four isolates, revealing extensive variation in biosynthetic repertoires despite a largely conserved proteomic background. Integration with MIBiG-associated predictions identified core GCFs showing similarity to characterized pathways associated with squalestatin S1, choline, scytalone/T3HN, cercosporin, and nectriapyrone biosynthesis. Additional characterized pathway-associated signatures showed partial or isolate-restricted distributions, including α-acorenol- and phyllostictine-associated BGCs in *D. inconspicua*, chaetolivacine, chrysogine-, patulin-, and AKML-associated BGCs in *D. vochysiae*, and enniatin-, azaphilone-, curvupallide-B-, aspyridone-, and wortmanamide-associated BGCs in *D*. sp. HANT25. Importantly, a substantial fraction of GCFs lacked significant correspondence to characterized MIBiG clusters, indicating considerable chemically uncharacterized biosynthetic potential. Collectively, these findings reveal a conserved genomic foundation coupled with extensive diversification of specialized-metabolite biosynthetic repertoires in medicinal plant-associated *Diaporthe* and identify conserved and isolate-restricted BGC families as candidates for future metabolomic and functional characterization.

## 1. Introduction

Endophytic fungi inhabit internal plant tissues for at least part of their life cycle without causing apparent disease and represent an important component of the plant-associated microbiome [1]. These fungi can influence plant fitness and ecological interactions while also producing structurally diverse specialized metabolites. Endophytes associated with medicinal plants are of particular interest because they constitute a promising reservoir of metabolites with antimicrobial, antioxidant, cytotoxic, and other potentially useful biological activities [2,3,4].

Among plant-associated fungi, the genus *Diaporthe* is notable for its ecological diversity and broad association with plants. Members of the genus occur as endophytes, saprobes, and pathogens, illustrating the ecological versatility of *Diaporthe*–plant interactions. Endophytic *Diaporthe* species have been recovered from numerous medicinal plants and are recognized as prolific producers of secondary metabolites. For example, endophytic *Diaporthe* isolates from *Catharanthus roseus* produce diverse volatile metabolites, particularly terpenoids, with inhibitory activity against several phytopathogenic fungi and oomycetes [5]. More recently, *Diaporthe* was among the predominant fungal genera isolated from the medicinal plant *Vochysia divergens* in the Brazilian Pantanal, and extracts from selected isolates exhibited substantial antifungal activity, further emphasizing the biotechnological potential of medicinal plant-associated *Diaporthe* [6].

The capacity of filamentous fungi to produce specialized metabolites is largely encoded by biosynthetic gene clusters (BGCs), in which genes for core biosynthetic enzymes are frequently colocated with genes encoding tailoring enzymes, transporters, and transcriptional regulators [7]. Major fungal BGC classes include those centered on polyketide synthases (PKSs), nonribosomal peptide synthetases (NRPSs), terpene biosynthetic enzymes, and hybrid biosynthetic systems [8,9]. Genome sequencing has revealed that filamentous fungi frequently encode substantially larger repertoires of BGCs than are reflected by metabolites detected under standard or limited cultivation conditions, because many biosynthetic pathways remain silent or weakly expressed under laboratory conditions [10,11,12]. Consequently, genome mining can reveal cryptic biosynthetic potential that is not apparent from conventional metabolite profiling and can provide a framework for prioritizing pathways for subsequent activation and functional characterization [10,12].

This genomic perspective is especially relevant to *Diaporthe*. Genome mining of *Diaporthe kyushuensis* ZMU-48-1 identified 98 BGCs, approximately 60% of which lacked significant similarity to known clusters; integration of genome mining with altered cultivation conditions subsequently revealed substantial chemical diversity, including previously undescribed metabolites [13]. Broader comparative genomic analyses have further indicated that secondary-metabolism genes constitute a particularly variable component of *Diaporthe* genomes and that the genus possesses an exceptionally extensive repertoire of specialized-metabolite BGCs. Chromosomal rearrangements, variation within BGCs, and horizontal gene transfer have been implicated in diversification of this biosynthetic arsenal [14]. Together, these observations suggest that comparative analysis at both the genome and BGC-family levels is necessary to distinguish conserved biosynthetic functions from lineage- or isolate-specific metabolic potential.

Despite increasing recognition of the metabolic richness of *Diaporthe*, comparatively little is known about how biosynthetic repertoires vary among endophytic isolates associated with medicinal plants. Most bioprospecting studies have focused on cultured metabolites or biological activity, whereas genome-scale comparisons can reveal pathways that are conserved, diversified, or unique even when their products have not yet been experimentally detected. Grouping related BGCs into gene cluster families (GCFs) provides an additional level of resolution by allowing homologous biosynthetic systems to be compared across genomes and by distinguishing broadly conserved pathways from isolate-specific and chemically uncharacterized biosynthetic families. Such information can help prioritize BGCs for subsequent metabolomic, expression, and functional studies.

In this study, we performed a comparative genomic investigation of four medicinal plant-associated endophytic *Diaporthe* isolates: *D. inconspicua* LGMF1612 from *Maytenus ilicifolia*, *D.* cf. *heveae* LGMF1633 and *D. vochysiae* LGMF1583 from *Vochysia divergens*, and *Diaporthe* sp. HANT25 from *Hydnocarpus anthelminthicus* [15,16,17,18]. These isolates were selected because they represent endophytic *Diaporthe* associated with medicinal plants for which genome assemblies and predicted proteomes were publicly available, enabling standardized comparative genomic and biosynthetic analyses. Their association with three different medicinal plant hosts, together with the occurrence of two taxonomically distinct isolates from *V. divergens*, also provided an opportunity to compare biosynthetic repertoires across different host associations as well as between isolates recovered from the same host species. The four isolates were therefore used as an available comparative dataset rather than as representatives of the full phylogenetic or ecological diversity of the genus *Diaporthe*. We compared their genome and proteome characteristics, orthologous protein repertoires, and functional profiles, with particular emphasis on functions related to transport, oxidation–reduction, detoxification, transcriptional regulation, and specialized metabolism. We further used genome mining and GCF analysis to characterize the diversity and conservation of BGCs, distinguish characterized metabolite-associated pathways from uncharacterized biosynthetic families, and identify isolate-specific biosynthetic signatures. This comparative framework provides insight into the shared and variable genomic architecture underlying specialized metabolism in medicinal plant-associated *Diaporthe* and identifies conserved and isolate-restricted BGCs that can guide future natural-product discovery and functional characterization.

## 2. Materials and Methods

### 2.1. Fungal Isolates and Genomic Datasets

Four endophytic *Diaporthe* isolates associated with medicinal plants were included in the comparative genomic analysis: *Diaporthe inconspicua* LGMF1612 (JGI genome ID: Diainc1), isolated from *Maytenus ilicifolia*; *Diaporthe* cf. *heveae* LGMF1633 (JGI genome ID: DiaLGMF1633) and *Diaporthe vochysiae* LGMF1583 (JGI genome ID: Diavoc1), both associated with *Vochysia divergens*; and *Diaporthe* sp. HANT25 (NCBI assembly accession: GCA_013435955.1), associated with *Hydnocarpus anthelminthicus*. The genome assemblies, structural annotations, and predicted protein sequences of *D. inconspicua* LGMF1612, *D.* cf. *heveae* LGMF1633, and *D. vochysiae* LGMF1583 were retrieved from the Joint Genome Institute (JGI) MycoCosm Data Portal (https://mycocosm.jgi.doe.gov/, accessed on 27 August 2026), whereas the corresponding genomic dataset of *Diaporthe* sp. HANT25 was obtained from NCBI GenBank. The repository-specific genome identifiers and assembly characteristics are summarized in Table 1, with additional genomic information provided in Table S1.

To establish the phylogenetic relationships of the four focal isolates within the broader diversity of *Diaporthe*, a multilocus phylogenetic analysis was performed using the internal transcribed spacer (ITS), β-tubulin (TUB2), histone H3 (HIS3), and translation elongation factor 1-α (TEF1) loci [19]. Homologous sequences from representative *Diaporthe* species were retrieved from publicly available sequence data, and the corresponding loci for the four focal isolates were extracted from their genome assemblies. The four marker sequences for each taxon were concatenated in the order ITS–TUB2–HIS3–TEF1. Sequence alignment and trimming of poorly aligned regions were performed using MEGA version 11. Maximum-likelihood phylogenetic reconstruction was conducted using the Tamura–Nei (TN93) nucleotide substitution model, assuming uniform evolutionary rates among sites. The concatenated alignment was analyzed as a single, unpartitioned dataset, with gaps and missing data handled using the “Use All Sites” option in MEGA11. Branch support was evaluated using 100 bootstrap replicates [20]. *Diaporthella corylina* CBS 121124 was included as the outgroup.

### 2.2. Comparative Proteome and Orthology Analysis

Predicted protein sequences from the four *Diaporthe* genomes were subjected to comparative orthology analysis using the OrthoVenn3 web platform [21]. Orthologous protein clustering was performed using the OrthoMCL algorithm with an E-value threshold of 1 × 10^−10^ and a Markov clustering inflation parameter of 1.20. All remaining parameters were maintained at their default settings. Orthologous clusters were categorized according to their distribution among the four isolates as core clusters (shared by all four isolates), clusters shared by three or two isolates, or isolate-specific clusters.

### 2.3. Functional Annotation of Predicted Proteins

Functional annotation of the four predicted proteomes of *Diaporthe* species was performed using eggNOG-mapper (version 3.0) [22]. Protein sequences were queried against the eggNOG orthology database, and functional assignments were retrieved for downstream comparative analysis. Pfam-domain annotations were extracted from the eggNOG-mapper output and summarized independently for each isolate. Domain frequencies were compared among the four proteomes to identify abundant functional groups potentially associated with ecological adaptation and specialized metabolism. GO terms assigned by eggNOG-mapper were compiled for each isolate and categorized according to the three principal GO domains: biological process, molecular function, and cellular component. Comparative GO profiles were generated to evaluate broad functional similarities and differences among the isolates. The functional annotation data are provided in Table S2.

### 2.4. Prediction and Characterization of Biosynthetic Gene Clusters

Biosynthetic gene clusters (BGCs) were identified independently in the four *Diaporthe* genomes using antiSMASH (version 8.0.4) in fungal mode with the detection strictness set to relaxed [23]. Other settings were maintained at their default values unless otherwise specified. The proteins encoded within the predicted BGC regions were extracted and subjected to Pfam domain analysis. Clusters containing more than one biosynthetic signature were retained according to the corresponding antiSMASH multiclass annotation. The number and distribution of BGC classes were compared among the four isolates to evaluate differences in their predicted specialized-metabolite biosynthetic capacity. Predicted BGCs were additionally compared with experimentally characterized biosynthetic pathways using the KnownClusterBlast module implemented in antiSMASH (version 8.0.4), using the MIBiG (version 4.0) reference dataset [24]. For each predicted BGC, the KnownClusterBlast output was examined to determine the percentage of genes in the matched MIBiG reference cluster showing significant similarity to genes in the query region. A study-defined operational cutoff of ≥45% reference-cluster gene coverage was applied consistently across all four genomes to retain candidate associations with characterized biosynthetic pathways while excluding lower-coverage matches. This criterion represents the proportion of genes in the matched MIBiG reference cluster with significant matches in the query BGC, as reported by antiSMASH KnownClusterBlast, rather than whole-cluster sequence identity. The 45% cutoff was selected specifically for the present study and is not a threshold prescribed or recommended by antiSMASH. Furthermore, meeting this criterion does not establish functional equivalence or confirm production of the corresponding metabolites. At the individual BGC level, BGCs with a retained MIBiG association meeting this criterion were designated as characterized pathway-associated BGCs, whereas BGCs lacking a retained MIBiG association were designated as chemically uncharacterized BGCs. These assignments indicate genomic similarity to characterized biosynthetic pathways and do not demonstrate pathway expression or production of the corresponding metabolite.

### 2.5. Gene Cluster Family Analysis

To compare BGC relatedness beyond direct similarity to characterized reference clusters, all antiSMASH-predicted BGCs from the four genomes were subjected to gene cluster family analysis using BiG-SLiCE (version 1.0) [25]. Individual antiSMASH region GenBank files were organized according to isolate and used as input. BGCs were grouped into GCFs using a distance threshold of 0.4, with eight computational threads. The distance threshold of 0.4 was selected as an operational cutoff to provide a consistent basis for grouping related BGCs across the four isolates while distinguishing differences in their biosynthetic feature profiles. BiG-SLiCE represents BGCs using biosynthetic protein-domain features and groups related clusters according to similarity in their biosynthetic feature profiles. GCFs were subsequently categorized according to their distribution among isolates. A GCF was considered core when at least one member occurred in each of the four isolates. GCFs represented in three or two isolates were classified as partially shared, whereas GCFs represented in only one isolate were designated isolate-restricted. Core GCF status therefore indicates shared GCF membership based on similarity in biosynthetic feature profiles and does not imply complete sequence conservation, conserved gene order, or strict orthology among the constituent BGCs. For GCF-level interpretation of characterized pathway associations, the MIBiG assignments of the constituent BGCs were considered. A GCF was classified as characterized pathway-associated when all constituent BGCs showed a retained association with the same characterized MIBiG pathway. GCFs in which a retained MIBiG association was detected in only a subset of constituent BGCs were classified as partially characterized pathway-associated, whereas GCFs in which none of the constituent BGCs showed a retained MIBiG association were classified as chemically uncharacterized. Selected BGCs representing shared, isolate-restricted, or characterized pathway-associated GCFs were comparatively visualized using clinker (version 0.28) [26].

## 3. Results

### 3.1. Genome Characteristics of the Four Diaporthe Isolates

Four medicinal plant-associated endophytic *Diaporthe* isolates, including *D. inconspicua* LGMF1612, *D.* cf. *heveae* LGMF1633, *D. vochysiae* LGMF1583, and *D*. sp. HANT25, were included in the comparative genomic analysis (Table 1). The four genomes showed moderate variation in genome size and assembly contiguity (Table 1). Genome sizes ranged from 52.45 Mb in *D.* cf. *heveae* LGMF1633 to 60.11 Mb in *D. vochysiae* LGMF1583, while GC content was relatively consistent, ranging from 51.68% to 52.73%. *D. inconspicua* LGMF1612 had a genome size of 56.26 Mb, whereas the *D.* sp. HANT25 genome was 55.34 Mb. Among the four assemblies, *D. vochysiae* LGMF1583 showed the highest N50 (8.38 Mb), whereas HANT25 had an N50 of 2.00 Mb.

**Figure 1:**
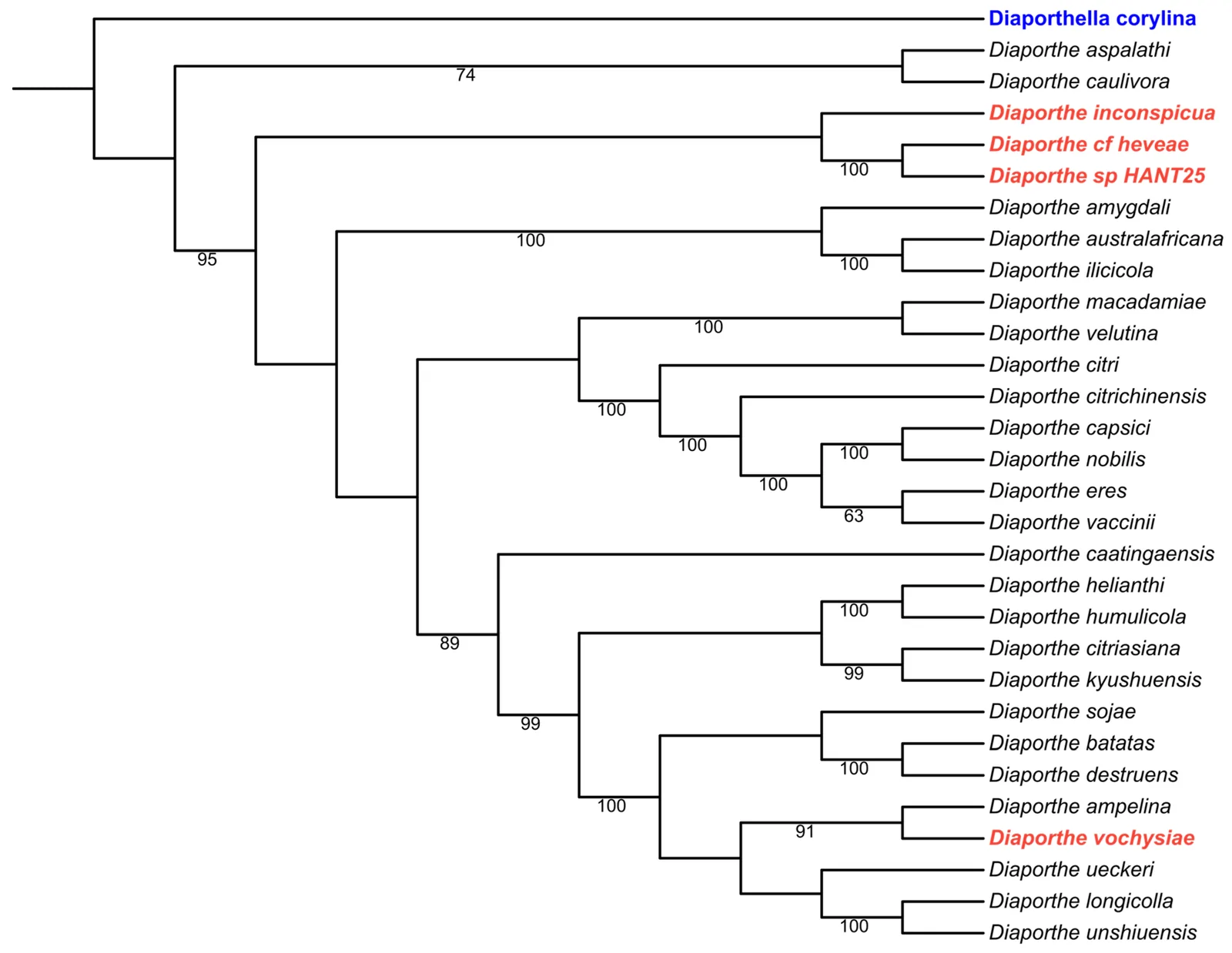
Multilocus phylogenetic relationships of the four medicinal plant-associated *Diaporthe* isolates and representative *Diaporthe* species. The phylogenetic tree was reconstructed from concatenated ITS, TUB2, HIS3, and TEF1 sequences. The four isolates analyzed in the present study are highlighted in red, and *Diaporthella corylina* CBS 121124, used as the outgroup, is shown in blue. Numbers at nodes indicate bootstrap support values (%).

Multilocus phylogenetic analysis based on concatenated ITS, TUB2, HIS3, and TEF1 sequences placed the four focal isolates at different positions within the sampled *Diaporthe* diversity (Figure 1). *D. inconspicua* LGMF1612, *D*. cf. *heveae* LGMF1633, and *Diaporthe* sp. HANT25 occurred within the same major lineage, with *D*. cf. *heveae* LGMF1633 and *Diaporthe* sp. HANT25 forming a strongly supported clade (100% bootstrap support). In contrast, *D. vochysiae* LGMF1583 occupied a separate position within the phylogeny, clustering with *D. ampelina*. *Diaporthella corylina* CBS 121124 was used as the outgroup. Thus, the four focal isolates encompass phylogenetically differentiated lineages within the sampled diversity of *Diaporthe*.

**Figure 2:**
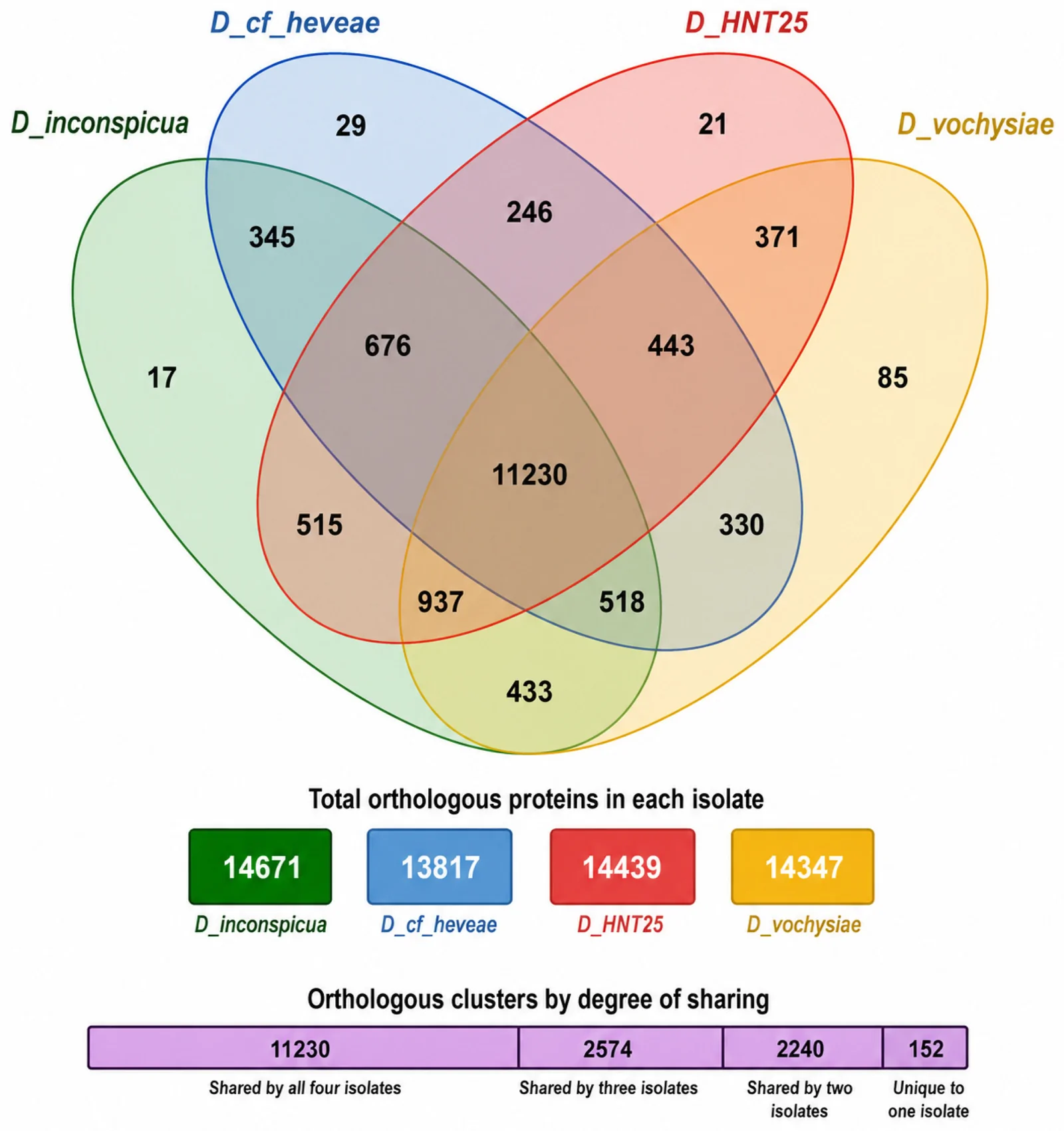
Comparative orthology of predicted proteins among four medicinal plant-associated endophytic *Diaporthe* isolates. Venn diagram showing shared and isolate-specific orthologous clusters identified using OrthoVenn3 in *D. inconspicua* LGMF1612, *D.* cf. *heveae* LGMF1633, *D. vochysiae* LGMF1583, and *D*. sp. HANT25. The central intersection represents 11,230 orthologous clusters shared by all four isolates; 2574 and 2240 clusters were shared by three and two isolates, respectively, whereas 152 clusters were isolate-specific.

Despite this phylogenetic differentiation, comparative orthology analysis revealed a substantial shared protein repertoire among the four isolates. A total of 16,196 orthologous clusters were identified, of which 11,230 (69.3%) were shared across all four isolates (Figure 2; Table S3). An additional 2574 and 2240 clusters were shared among three and two isolates, respectively, whereas only 152 clusters (0.9%) were isolate-restricted. *D. vochysiae* LGMF1583 contained the largest isolate-restricted component (85 clusters), followed by *D*. cf. *heveae* LGMF1633 (29), *Diaporthe* sp. HANT25 (21), and *D. inconspicua* LGMF1612 (17). In addition, 2404, 1875, 1714, and 1751 unclustered singleton proteins were identified in *D. vochysiae*, *D*. cf. *heveae*, *Diaporthe* sp. HANT25, and *D. inconspicua*, respectively. Overall, the four isolates therefore exhibited a largely shared proteomic background accompanied by smaller partially shared, isolate-restricted, and unclustered components.

### 3.2. Functional Features Associated with Ecological Adaptation and Specialized Metabolism

**Figure 3:**
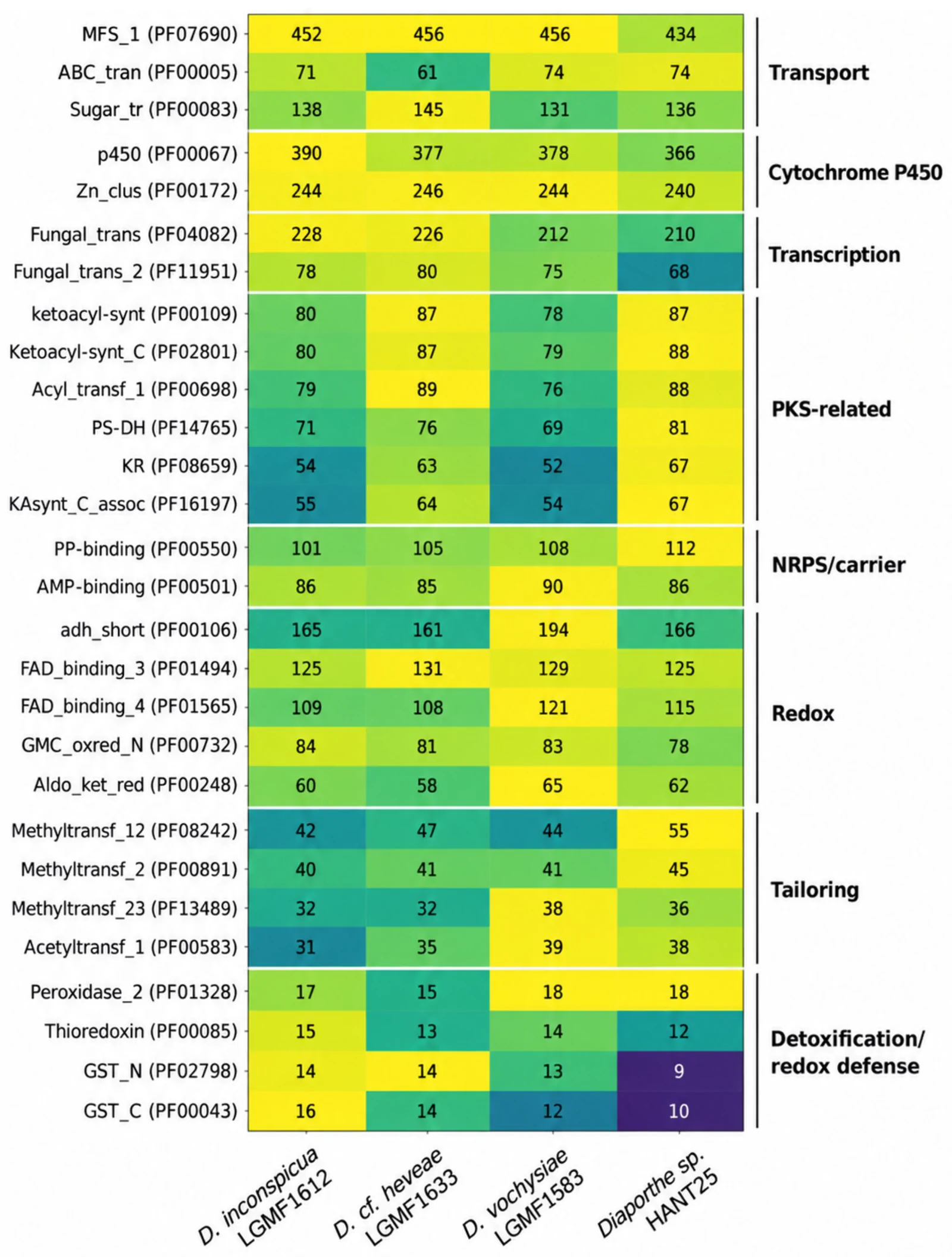
Distribution of protein domains associated with transport, transcriptional regulation, and specialized metabolism in four endophytic *Diaporthe* isolates. Values within cells indicate the number of proteins containing each Pfam domain in *D. inconspicua* LGMF1612, *D*. cf. *heveae* LGMF1633, *D. vochysiae* LGMF1583, and *Diaporthe* sp. HANT25.

Comparative Pfam analysis revealed broadly similar domain profiles across the four *Diaporthe* proteomes, with prominent representation of domains associated with membrane transport, transcriptional regulation, oxidation–reduction, detoxification, and specialized-metabolite biosynthesis (Figure 3). Transport-related domains were particularly abundant. The major facilitator superfamily domain MFS_1 (PF07690) was detected in 452, 456, 456, and 434 proteins in *D. inconspicua* LGMF1612, *D.* cf. *heveae* LGMF1633, *D. vochysiae* LGMF1583, and *D*. sp. HANT25, respectively. Sugar_tr (PF00083) occurred in 131–145 proteins, while the ABC transporter ATP-binding domain ABC_tran (PF00005) was present in 61–74 proteins. Transcription-associated domains were also abundant, including Zn_clus (PF00172; 240–246 proteins), Fungal_trans (PF04082; 210–228), and Fungal_trans_2 (PF11951; 68–80). The p450 (PF00067) domain represented one of the most abundant functional domains, occurring in 366–390 proteins across the four isolates.

Multiple domains associated with oxidation–reduction and detoxification were consistently detected (Figure 3). The short-chain dehydrogenase/reductase domain adh_short (PF00106) occurred in 161–194 proteins, while FAD_binding_3 (PF01494) and FAD_binding_4 (PF01565) were present in 125–131 and 108–121 proteins, respectively. GMC_oxred_N (PF00732) occurred in 78–84 proteins and Aldo_ket_red (PF00248) in 58–65 proteins. Additional redox- and detoxification-associated domains included Peroxidase_2 (PF01328), Thioredoxin (PF00085), and the glutathione S-transferase domains GST_N (PF02798) and GST_C (PF00043).

The four proteomes also contained numerous domains associated with specialized-metabolite biosynthesis (Figure 3). PKS-associated domains included ketoacyl-synt (PF00109; 78–87 proteins), Ketoacyl-synt_C (PF02801; 79–88), Acyl_transf_1 (PF00698; 76–89), PS-DH (PF14765; 69–81), KR (PF08659; 52–67), and KAsynt_C_assoc (PF16197; 54–67). The carrier-protein-associated PP-binding (PF00550) domain occurred in 101–112 proteins, whereas AMP-binding (PF00501) was detected in 85–90 proteins. Domains representing potential tailoring functions were also present in all four isolates, including Methyltransf_12 (PF08242; 42–55 proteins), Methyltransf_2 (PF00891; 40–45), Methyltransf_23 (PF13489; 32–38), and Acetyltransf_1 (PF00583; 31–39).

**Figure 4:**
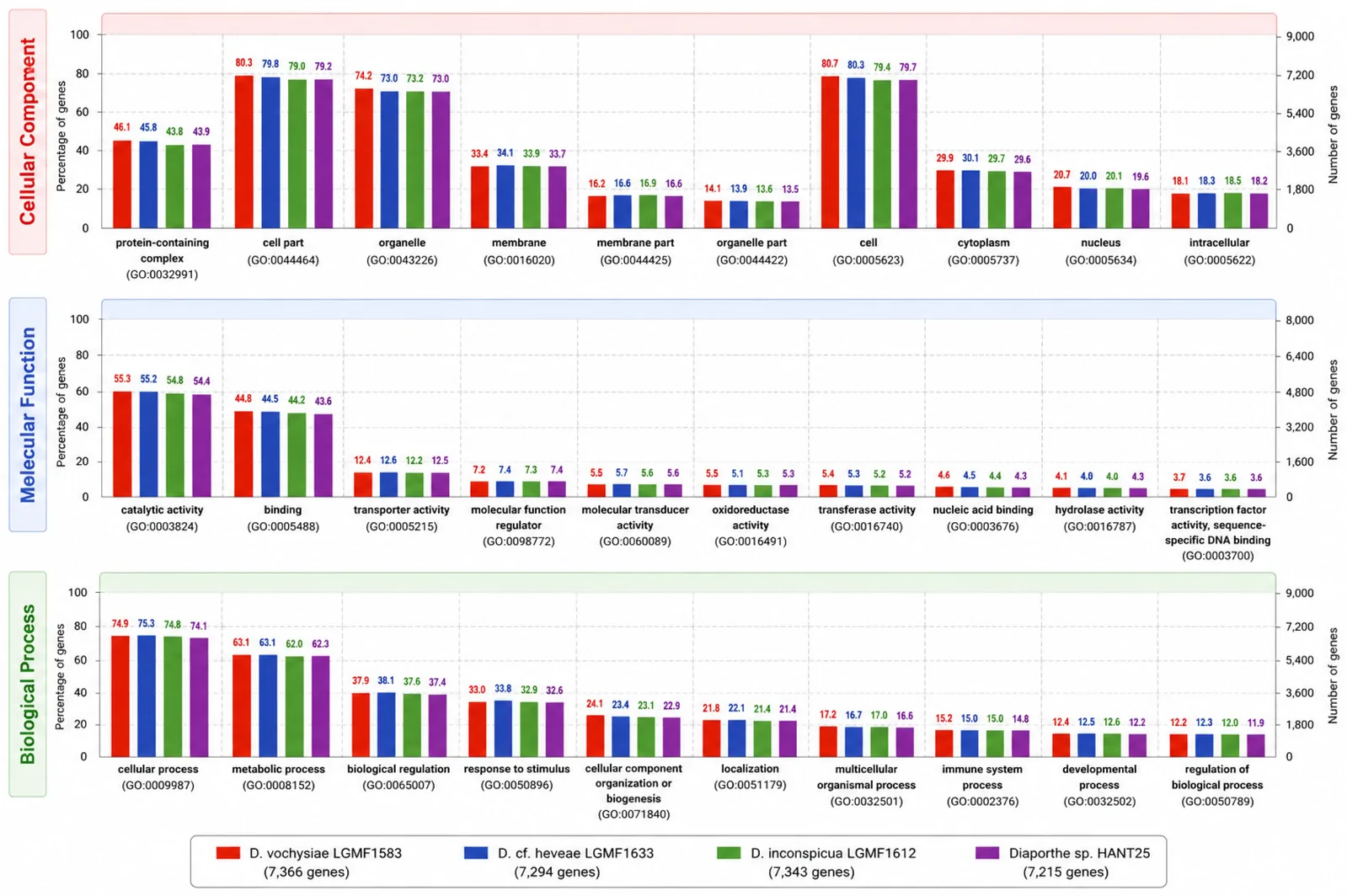
Gene Ontology (GO) classification of functionally annotated proteins in four endophytic *Diaporthe* isolates. The bar plots show the distribution of proteins assigned to major GO terms within the three principal ontologies: Cellular Component, Molecular Function, and Biological Process. Bars represent *D. vochysiae* LGMF1583, *D*. cf. *heveae* LGMF1633, *D. inconspicua* LGMF1612, and *Diaporthe* sp. HANT25. Values above the bars indicate the percentage of GO-annotated genes assigned to each term. GO accession numbers are provided below the corresponding functional categories. The bar colors represent the four isolates: red, *D. vochysiae* LGMF1583; blue, *D*. cf. *heveae* LGMF1633; green, *D. inconspicua* LGMF1612; and purple, *Diaporthe* sp. HANT25.

Gene Ontology classification showed highly comparable functional distributions among the four isolates (Figure 4). Within the Biological Process category, cellular process was the most represented term (75%), followed by metabolic process (63%), biological regulation (38%), and response to stimulus (34%). Molecular Function annotations were dominated by catalytic activity (55%) and binding (45%), with transporter activity accounting for approximately 12–13% of annotated proteins. Within Cellular Component, cell and cell part were the predominant categories (80%), followed by organelle (72%) and membrane-associated categories.

Together, the targeted functional categories showed extensive representation of transport, transcriptional regulation, oxidation–reduction, detoxification, cytochrome P450, and specialized-metabolism-associated proteins in all four isolates. Among the examined domains, MFS transporters, cytochrome P450s, fungal transcriptional regulators, and redox enzymes were particularly numerous, while PKS-, NRPS/carrier-protein-, and tailoring-enzyme-associated domains formed a substantial component of the specialized-metabolism-related repertoire.

### 3.3. Distribution and Diversity of Biosynthetic Gene Clusters

**Figure 5:**
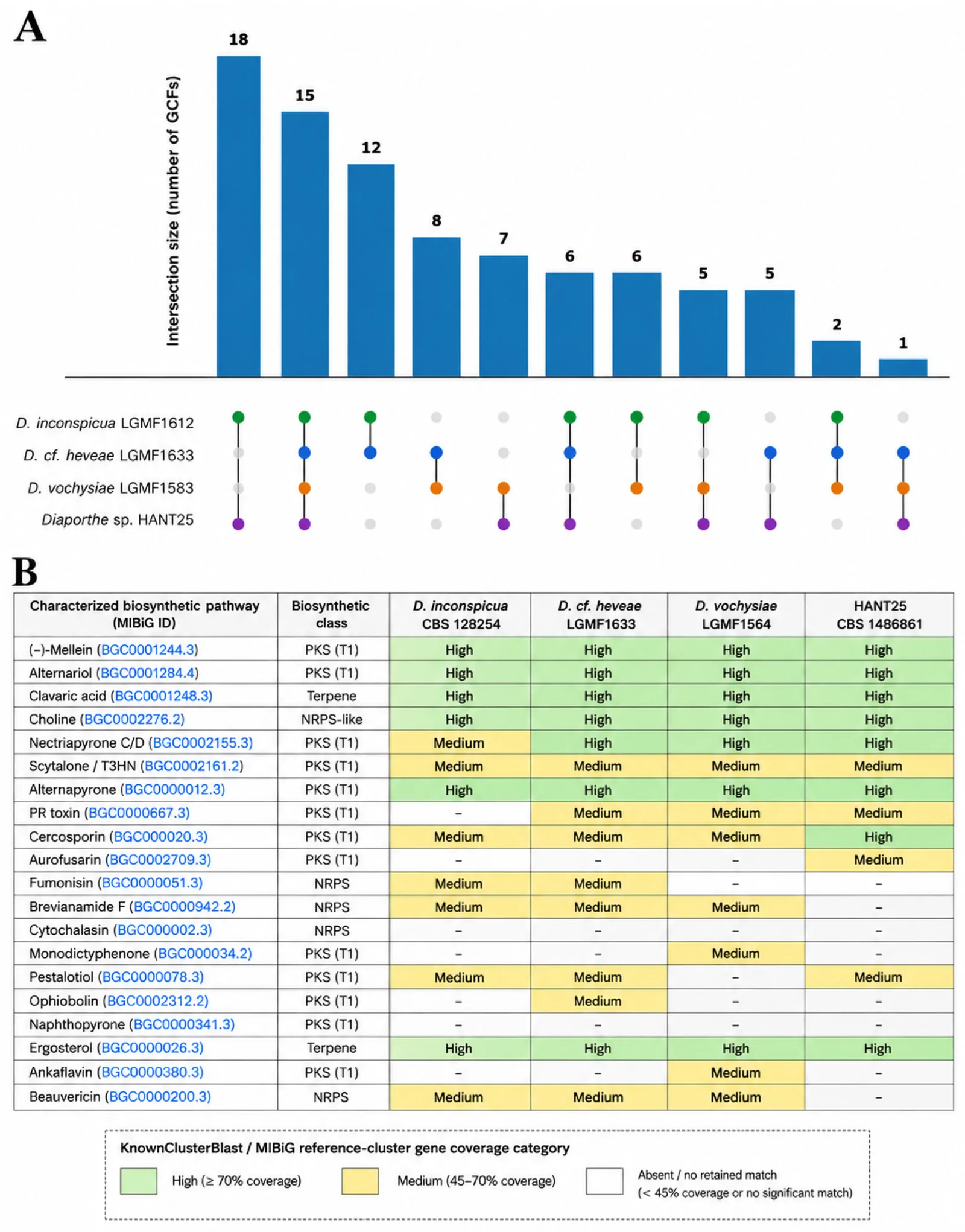
Distribution and characterized biosynthetic potential of gene cluster families (GCFs) across four endophytic *Diaporthe* isolates. (**A**) UpSet plot showing the distribution of GCFs shared among *D. inconspicua* LGMF1612, *D.* cf. *heveae* LGMF1633, *D. vochysiae* LGMF1583, and *Diaporthe* sp. HANT25. Bars indicate the number of GCFs corresponding to each isolate combination, with connected colored dots indicating the isolates represented in each intersection. (**B**) Heatmap showing representative biosynthetic pathway associations identified by antiSMASH KnownClusterBlast/MIBiG analysis across the four isolates. Colors indicate reference-cluster gene coverage categories: high (≥70%) and medium (≥45% to <70%). White cells indicate the absence of a retained MIBiG association under the operational ≥45% reference-cluster gene coverage threshold used in this study. “-” indiacates absent of biostnetic cluster.

Genome mining using antiSMASH identified 481 biosynthetic gene clusters (BGCs) across the four *Diaporthe* genomes, with 112–129 BGCs per isolate (Table 2 and Table S4; Figure 5). *D*. sp. HANT25 contained the largest repertoire with 129 BGCs, followed by *D.* cf. *heveae* LGMF1633 (123), *D. inconspicua* LGMF1612 (117), and *D. vochysiae* LGMF1583 (112). Overall, the four isolates showed broadly similar BGC-class compositions but differed in the relative representation of specific biosynthetic classes. T1PKS-associated regions were consistently the most abundant across all four genomes, while the clearest differences occurred in terpene- and NRPS-associated repertoires. D. cf. *heveae* LGMF1633 showed the highest number of terpene-associated regions, whereas D. *vochysiae* LGMF1583 contained the highest number of NRPS-associated regions. Thus, the principal differences among the isolates reflected shifts in the relative representation of particular biosynthetic classes rather than major changes in overall BGC-class composition.

The BGC repertoires comprised multiple biosynthetic classes, with type I polyketide synthase (T1PKS) clusters representing the most abundant class in all four genomes (Table 2). T1PKS-associated regions numbered 53, 57, 54, and 58 in *D. inconspicua*, *D.* cf. *heveae*, *D. vochysiae*, and *D*. sp. HANT25, respectively. NRPS clusters were also prevalent, ranging from 17 to 25, with the highest number in *D. vochysiae* (25), followed by *D*. sp. HANT25 (24), *D. inconspicua* (22), and *D.* cf. *heveae* (17). NRPS-like clusters ranged from 18 to 22, whereas terpene-associated clusters showed greater variation, ranging from 24 in *D. vochysiae* to 40 in *D.* cf. *heveae*. Indole-associated BGCs occurred at lower frequencies, with 6–9 clusters per genome. T3PKS, fungal RiPP, terpene-precursor, β-lactone, isocyanide, siderophore, and other minor BGC classes were also detected at comparatively low frequencies (Table 2 and Table S4).

A substantial proportion of the predicted regions contained more than one biosynthetic class, indicating the presence of hybrid/multiclass BGCs (Figure 5). Based on mutually exclusive region classification, hybrid/multiclass clusters accounted for 24, 28, 21, and 23 BGCs in *D. inconspicua*, *D.* cf. *heveae*, *D. vochysiae*, and *D*. sp. HANT25, respectively. Overall, the antiSMASH profiles showed extensive BGC repertoires in all four isolates, with a consistent predominance of polyketide-associated biosynthetic machinery together with substantial NRPS, NRPS-like, terpene, and hybrid cluster components (Table 2 and Table S4; Figure 5).

### 3.4. Shared Biosynthetic Pathways and Their GCF Organization Across Diaporthe Isolates

Integration of antiSMASH KnownClusterBlast/MIBiG predictions with BiG-SLiCE clustering revealed different patterns of shared GCF membership among characterized pathway-associated BGCs across the four endophytic *Diaporthe* isolates (Table S5). Of the 348 GCFs, 15 were classified as core GCFs, with at least one BGC member represented in each of the four isolates (Figure 5A, Table S5). Five of these core families-GCF84, GCF109, GCF197, GCF214, and GCF241 showed similarity to the same characterized MIBiG-associated pathway in all four genomes, corresponding to squalestatin S1, choline, scytalone/T3HN, cercosporin, and nectriapyrone-associated BGCs, respectively (Figure 5B; Table S6). Additional characterized pathways were detected in all four isolates but were distributed among different GCFs.

**Figure 6:**
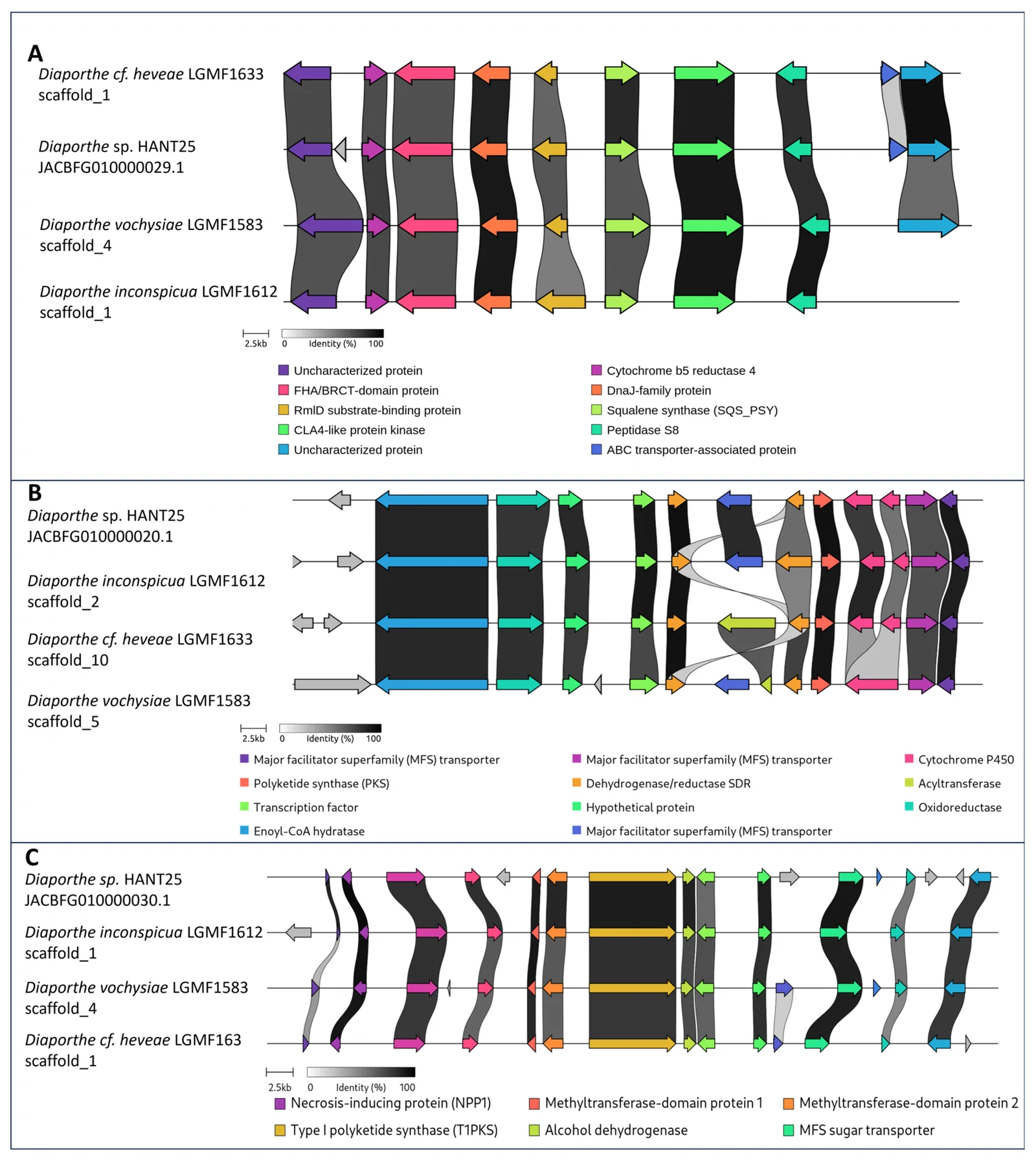
Comparative synteny and gene organization of shared metabolite-associated biosynthetic gene cluster families across four endophytic *Diaporthe* isolates. (**A**) Squalestatin S1-associated GCF84, (**B**) cercosporin-associated GCF214, and (**C**) nectriapyrone C/D-associated GCF241. Gene arrows indicate gene position, orientation, and predicted function, while connecting regions represent sequence similarity between homologous loci. Darker shading indicates higher sequence identity. The scale bar represents 2.5 kb.

The squalestatin S1-associated terpene BGC (MIBiG accession BGC0001839.3) was consistently assigned to GCF84 across all four isolates. The corresponding BGCs contained genes encoding a squalene synthase with an SQS_PSY domain and a DnaJ-family protein containing DnaJ and DnaJ-X domains (Figure 6A). Similarly, GCF214 represented a core T1PKS GCF associated with the cercosporin biosynthetic pathway (MIBiG accession BGC0001541.3) and contained a CTB1-like polyketide synthase together with O-methyltransferase-, laccase-, and transporter-associated components (Figure 6B). Another characterized core family, GCF241, comprised T1PKS BGCs associated with nectriapyrone C/D/nectriapyrone biosynthesis (MIBiG accession BGC0002155.3) and contained a multidomain type I PKS together with two methyltransferase-associated proteins (Figure 6C). In addition, the choline-associated NRPS-like BGC (MIBiG accession BGC0002276.2) and the scytalone/T3HN-associated T1PKS BGC (MIBiG accession BGC0002161.2) were represented by the core GCF109 and GCF197, respectively, across all four genomes. Collectively, these core GCFs represent a shared core of characterized pathway-associated biosynthetic potential among the four *Diaporthe* isolates despite substantial variation in their overall BGC repertoires.

Several additional characterized pathways were detected in all four isolates but were distributed among multiple GCFs (Table S5). The (−)-mellein-associated pathway (BGC0001244.3) was represented by T1PKS BGCs. These BGCs were distributed among GCF208, GCF237, and GCF319. Likewise, alternariol-associated T1PKS BGCs (BGC0001284.4) were identified in all four genomes but were assigned to GCF200, GCF203, GCF295, and GCF193, respectively.

Characterized pathway-associated GCFs represented in three isolates formed a second level of conservation, comprising three GCFs (Table S5). GCF22, shared by *D. inconspicua* LGMF1612, *D. vochysiae* LGMF1583, and *D.* sp. HANT25, was associated with the clavaric acid pathway (BGC0001248.3). GCF278, shared by *D.* cf. *heveae* LGMF1633, *D. vochysiae* LGMF1583, and HANT25, showed consistent similarity to the alternapyrone pathway (BGC0000012.3) across all three member BGCs. GCF303, shared by *D. inconspicua*, *D*. cf. *heveae*, and *D*. sp. HANT25, was associated with the duclauxin pathway (BGC0001578.3). However, this family was classified as partially characterized pathway-associated because only two of its three constituent BGCs showed a retained MIBiG association.

Among characterized pathways shared by two isolates, 19 GCFs showed associations with characterized MIBiG clusters (Table S5). For example, high-confidence associations included GCF108, shared by *D. inconspicua* LGMF1612 and *D. vochysiae* LGMF1583, with ochrindole A (BGC0001712.3); GCF118, shared by *D. inconspicua* and *D.* cf. *heveae* LGMF1633, with pimara-8(14),15-diene (BGC0000673.3); GCF126, shared by *D. inconspicua* and HANT25, with hexadehydroastechrome/terezine-D/astechrome (BGC0000372.5); GCF167, shared by *D.* cf. *heveae* and *D*. sp. HANT25, with huperzine A (BGC0000812.3); GCF206, shared by *D.* cf. *heveae* and *D. vochysiae*, with duclauxin (BGC0001578.3); GCF208, shared by *D. inconspicua* and *D*. sp. HANT25, with (−)-mellein (BGC0001244.3); GCF221, shared by *D. inconspicua* and *D.* cf. *heveae*, with betaenones A–C (BGC0001264.3); GCF285, shared by *D. inconspicua* and *D. vochysiae*, with stipitatic acid (BGC0000154.4); and GCF294 and GCF315, shared by *D.* cf. *heveae* and *D*. sp. HANT25 and *D. inconspicua* and *D.* cf. *heveae*, respectively, with the zearalenone pathway (BGC0001057.4).

### 3.5. Isolate-Specific Characterized Pathway-Associated BGCs

**Figure 7:**
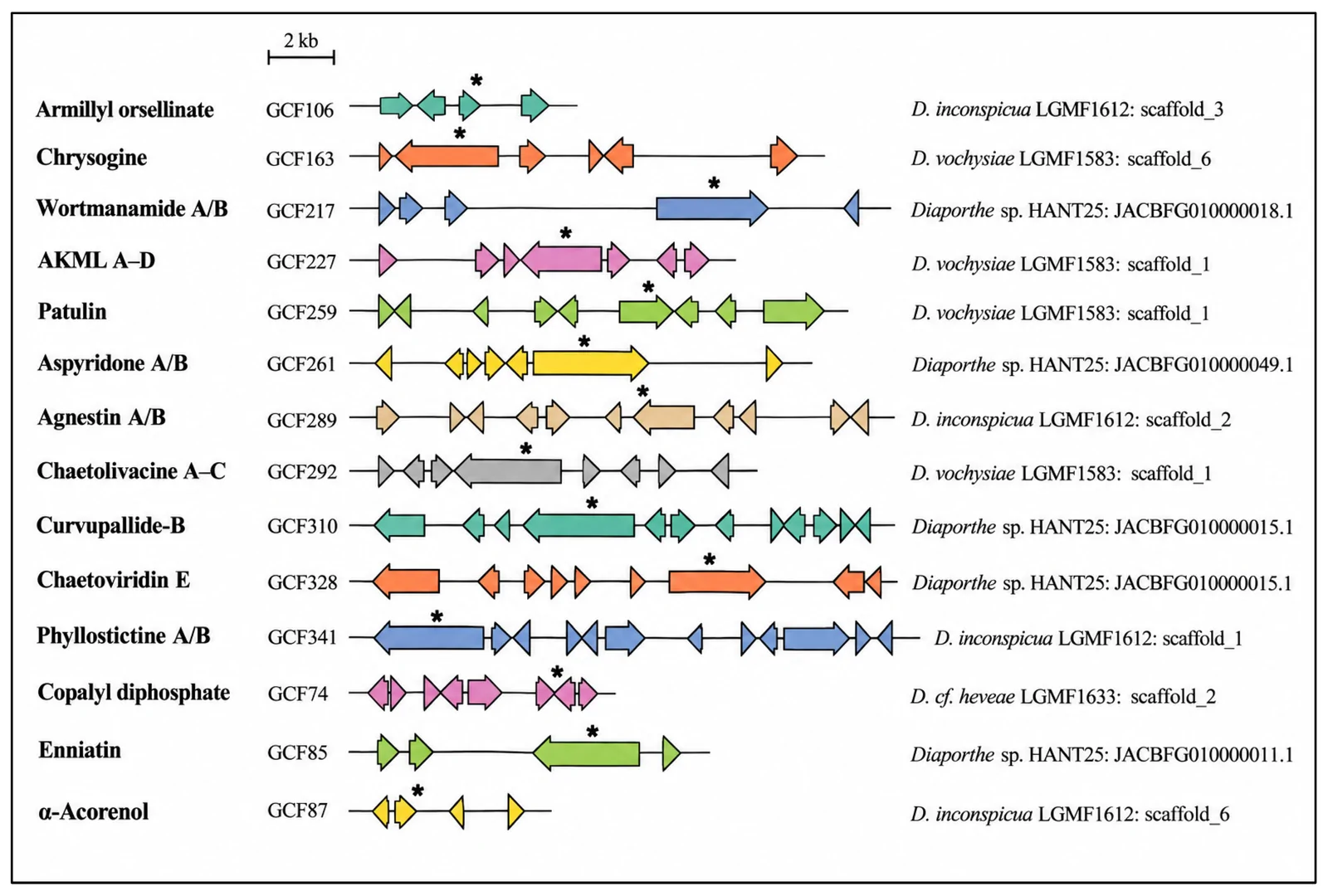
Isolate-specific characterized pathway-associated biosynthetic gene clusters (BGCs) identified across four endophytic *Diaporthe* isolates. Gene-cluster maps show BGCs associated with characterized metabolite pathways based on antiSMASH KnownClusterBlast/MIBiG analysis. Arrows indicate gene position and transcriptional orientation. Asterisks (*) indicate predicted key biosynthetic genes, identified based on their functional annotations. Detailed annotations of the genes within the predicted BGCs are provided in Table S4. These assignments are based on predicted gene functions and do not indicate experimental validation. The corresponding GCF, isolate, and genomic scaffold are indicated for each cluster. The scale bar represents 2 kb.

Beyond the characterized pathways shared among isolates, the isolate-specific BGC repertoire showed clear differences in both pathway diversity and biosynthetic class composition (Figure 7; Table S5). A total of 75 characterized pathway-associated GCFs were restricted to individual isolates, comprising 17 in *D. inconspicua* LGMF1612, 19 in *D.* cf. *heveae* LGMF1633, 22 in *D. vochysiae* LGMF1583, and 17 in *Diaporthe* sp. HANT25. An additional partially characterized pathway-associated GCF was restricted to *Diaporthe* sp. HANT25. The *D. inconspicua* repertoire included terpene, T1PKS, and hybrid NRPS–T1PKS pathways, while *D. vochysiae* exhibited NRPS, T1PKS, terpene–T1PKS, and NRPS–T1PKS associations. In contrast, selected BGCs from HANT25 included NRPS, T1PKS, and PKS–NRPS pathways, including two azaphilone-associated BGCs within GCF328. The *D.* cf. *heveae* repertoire included a copalyl diphosphate-associated terpene GCF. These isolate-restricted pathway-associated signatures highlight differences in the predicted biosynthetic potential of the four genomes. Representative BGCs illustrating these differences are described below.

In *D. inconspicua* LGMF1612, an isolate-specific terpene BGC assigned to GCF87 showed high-confidence similarity to the α-acorenol pathway (BGC0001481.3). The characterized-cluster match included a gene encoding a terpene-associated biosynthetic component. A second isolate-specific terpene family, GCF106, showed medium-confidence similarity to the armillyl orsellinate/8α-hydroxy-6-protoilludene pathway (BGC0002445.3) and contained the matched genes encoding cytochrome P450-associated components. In addition, the NRPS–T1PKS hybrid GCF341 showed medium-confidence similarity to the phyllostictine A/B pathway (BGC0001741.3). The matched genes encoded a PKS–NRPS hybrid backbone protein together with FAD-dependent oxidoreductase-, NADP-dependent dehydrogenase/enoyl-reductase-, cytochrome P450-, and SAM-dependent O-methyltransferase-associated proteins. An isolate-specific T1PKS family, GCF289, additionally showed similarity to the agnestin A/B biosynthetic pathway (Table S5). The isolate-specific characterized repertoire of *D.* cf. *heveae* LGMF1633 was distinguished by a terpene BGC assigned to GCF74 with medium-confidence similarity to the copalyl diphosphate pathway (BGC0000688.4). The corresponding cluster contained a terpene-cyclase-associated biosynthetic component consistent with diterpene scaffold formation.

*D. vochysiae* LGMF1583 contained the largest set of distinctive characterized pathway signatures. The NRPS–T1PKS hybrid GCF292 showed high-confidence similarity to the chaetolivacine A–C pathway (BGC0002538). A second isolate-specific family, GCF163, comprised an NRPS BGC showing high-confidence similarity to the chrysogine pathway (BGC0001545.3). A distinct terpene–T1PKS BGC assigned to GCF259 in *D. vochysiae* showed medium-confidence similarity to the patulin pathway (BGC0000120.5). The matched biosynthetic components included 6-methylsalicylic acid synthase, 6-methylsalicylic acid decarboxylase, m-hydroxybenzyl alcohol hydroxylase, m-cresol hydroxylase, isoamyl alcohol oxidase, and an ABC transporter, representing multiple biosynthetic and transport functions of the characterized pathway. In addition, the isolate-specific T1PKS GCF227 showed medium-confidence similarity to the AKML A–D pathway (BGC0002227.3) and retained the associated polyketide synthase component.

*D*. sp. HANT25 exhibited a distinct set of isolate-specific characterized NRPS, T1PKS, and hybrid BGCs. Most notably, GCF85 comprised an NRPS BGC with high-confidence similarity to the enniatin pathway (BGC0000342.4). The BGC contained a gene encoding an enniatin synthetase, providing a direct biosynthetic link to the characterized NRPS pathway. *D*. sp. HANT25 also contained an isolate-specific T1PKS family, GCF328, represented by BGCs showing similarity to characterized azaphilone pathways. One member showed similarity to the harziphilone/t22azaphilone/isoharziphilone pathway (BGC0002206.2) and contained a polyketide synthase together with a salicylate hydroxylase, MFS transporter, and Zn(II)Cys transcription factor. Another member of the same GCF showed similarity to the chaetoviridin E/11-epichaetomugilin A pathway (BGC0001405.4), indicating the presence of related azaphilone-associated BGC architectures within *D*. sp. HANT25.

Two isolate-specific PKS–NRPS hybrid families further distinguished *D*. sp. HANT25. GCF310 showed medium-confidence similarity to the curvupallide-B pathway (BGC0001563.3). Its matched gene complement included a PKS–NRPS hybrid backbone protein, together with O-methyltransferase, cytochrome P450, FAD- and NAD-dependent oxidoreductase, SDR-type oxidoreductase, monooxygenase, transcription-factor, and MFS-transporter-associated proteins. Similarly, GCF261 showed medium-confidence similarity to the aspyridone A/B pathway (BGC0000959.5) and contained a hybrid PKS–NRPS, cytochrome P450 monooxygenase, enoyl reductase, FAD-dependent monooxygenase, and exporter-associated component. An additional isolate-specific T1PKS family, GCF217, showed medium-confidence similarity to the wortmanamide A/B pathway (BGC0001954.2).

### 3.6. Distribution and Isolate Specificity of Uncharacterized Biosynthetic Gene Cluster Families

**Figure 8:**
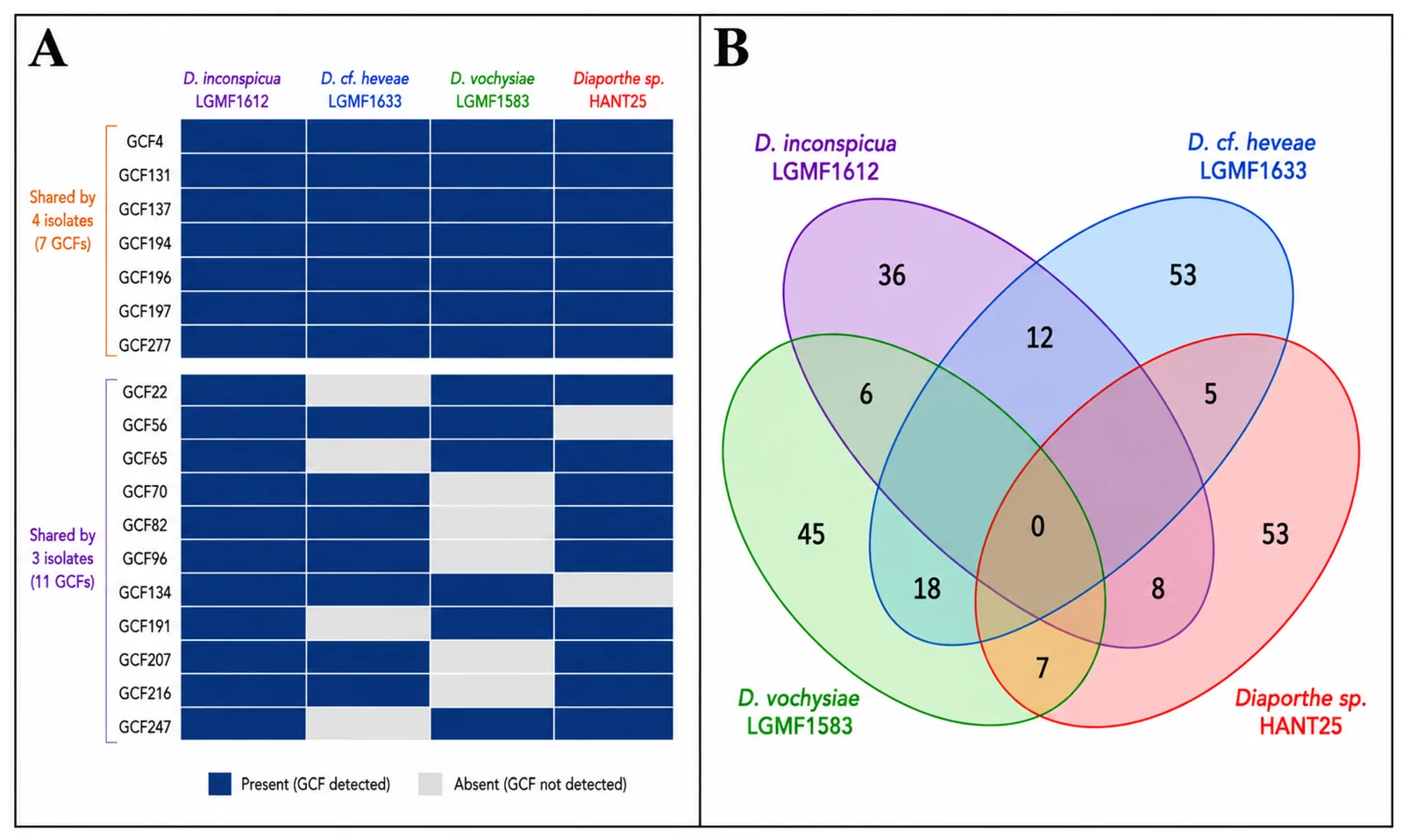
Distribution and isolate specificity of chemically uncharacterized biosynthetic gene cluster families (GCFs) among four medicinal plant-associated endophytic *Diaporthe* isolates. (**A**) Presence–absence heatmap showing seven core GCFs represented across all four isolates and 11 GCFs shared by three isolates. Dark blue indicates GCF presence, whereas light gray indicates absence. (**B**) Four-set Venn diagram illustrating the distribution of 187 isolate-restricted and 37 pairwise-shared chemically uncharacterized GCFs. Numbers in non-overlapping regions indicate isolate-restricted GCFs, whereas numbers in pairwise intersections indicate GCFs shared exclusively by two isolates. Together, both panels represent 242 chemically uncharacterized GCFs identified across the four isolates (Table S5).

Of the 348 GCFs identified across the four *Diaporthe* isolates, 91 (26.1%) were classified as characterized pathway-associated GCFs, 15 (4.3%) as partially characterized pathway-associated GCFs, and 242 (69.5%) as chemically uncharacterized GCFs (Table S5). These classifications were based on the retained MIBiG associations of their constituent BGCs, as described in Section 2.5. The chemically uncharacterized GCFs exhibited different patterns of sharing among isolates, ranging from families represented across all four isolates to those shared by three or two isolates and families restricted to a single isolate (Figure 8A,B). They encompassed multiple biosynthetic classes, including T1PKS, NRPS, NRPS-like, terpene, hybrid, indole, and fungal RiPP-associated BGCs.

Among the seven chemically uncharacterized core GCFs represented in all four isolates, none of the constituent BGCs showed a retained characterized MIBiG association. These core but uncharacterized families included BGCs with recognizable biosynthetic machinery but without sufficient KnownClusterBlast similarity to assign a specific metabolite. In particular, core families containing PKS-, NRPS/NRPS-like-, and terpene-associated core enzymes demonstrated that part of the shared biosynthetic repertoire remains chemically unassigned. In addition, four core GCFs were classified as partially characterized pathway-associated because a retained characterized-cluster match occurred in only a subset of their constituent BGCs. These families were not considered chemically uncharacterized, and the corresponding metabolite association was not extended to the remaining members solely on the basis of GCF membership.

A second group of 11 chemically uncharacterized GCFs was shared by three isolates (Table S5). These families retained similar biosynthetic feature profiles in three genomes but lacked a significant common MIBiG assignment. Their BGCs included PKS-, NRPS-like, terpene, and hybrid-type systems, with shared occurrence of the principal biosynthetic enzymes accompanied by variable complements of tailoring, regulatory, and transporter-associated genes. The absence of the corresponding GCF from the fourth isolate distinguished these families from the core chemically uncharacterized component.

Additional chemically uncharacterized families were distributed between two isolates (Table S5). A total of 37 GCFs showed this pairwise-shared distribution. These pairwise-shared GCFs represented a broader range of BGC architectures and included both single-class and hybrid biosynthetic systems. Several contained multidomain type I PKSs with combinations of KS, AT, ACP/phosphopantetheine-binding, KR, DH, and methyltransferase domains, whereas NRPS and NRPS-like families contained adenylation/AMP-binding and phosphopantetheine-binding domains, with condensation- or reductase-associated domains present in some clusters. Hybrid families combined PKS and NRPS biosynthetic modules within the same BGC. Despite the presence of these recognizable biosynthetic features, no common characterized metabolite could be assigned to these GCFs from the KnownClusterBlast/MIBiG analysis.

The greatest diversification occurred among isolate-restricted chemically uncharacterized GCFs. A total of 187 chemically uncharacterized GCFs were isolate-restricted. These families were represented by BGCs from only one of the four genomes and lacked significant characterized-cluster assignments (Table S5). Isolate-restricted uncharacterized clusters occurred in all four *Diaporthe* genomes and included T1PKS, NRPS, NRPS-like, terpene, and hybrid BGCs, together with less abundant indole- and fungal RiPP-associated systems. Many retained complete core biosynthetic proteins despite the absence of a recognizable characterized pathway, distinguishing them from regions for which biosynthetic class assignment itself was uncertain.

The uncharacterized T1PKS GCFs were particularly prominent and encoded diverse PKS domain architectures. In addition to the shared KS and AT domains, individual proteins differed in the presence of DH, KR, methyltransferase, and ACP/phosphopantetheine-binding domains. Uncharacterized NRPS and NRPS-like clusters similarly varied in their modular composition, while several isolate-restricted hybrid clusters encoded both PKS and NRPS catalytic machinery together with oxidoreductases, cytochrome P450s, methyltransferases, transporters, and transcriptional regulators. These differences further distinguished the uncharacterized BGCs at both core-enzyme and cluster-composition levels.

In addition to the 242 chemically uncharacterized GCFs, 15 GCFs were classified as partially characterized pathway-associated because only a subset of their constituent BGCs showed a retained MIBiG association (Table S5). These comprised four core GCFs, one GCF shared by three isolates, nine GCFs shared by two isolates, and one isolate-restricted GCF. For these families, characterized pathway associations were restricted to the supporting BGC members and were not extrapolated to other members solely on the basis of GCF membership.

Thus, the GCF analysis resolved the chemically uncharacterized biosynthetic repertoire into a hierarchical distribution comprising seven core GCFs represented in all four isolates, 11 families shared by three isolates, 37 families shared by two isolates, and 187 isolate-restricted families. Importantly, these GCFs were retained as uncharacterized biosynthetic families rather than assigned metabolite identities from biosynthetic class or GCF similarity alone. GCFs containing MIBiG support in only a subset of their members were instead retained as partially characterized pathway-associated GCFs. Together with the characterized pathways described in Section 3.4 and Section 3.5, this unassigned GCF component accounts for a substantial portion of the biosynthetic diversity encoded by the four medicinal plant-associated endophytic *Diaporthe* genomes.

## 4. Discussion

The four medicinal plant-associated endophytic *Diaporthe* isolates exhibit a broadly shared proteomic background but markedly more heterogeneous biosynthetic repertoires. This contrast suggests that specialized-metabolite pathways constitute a comparatively flexible component of these genomes, potentially contributing to differences in chemical interactions despite substantial conservation of general cellular functions. Because GCF membership reflects similarity in biosynthetic features rather than strict sequence or synteny conservation, the patterns observed here are best interpreted as differences in predicted biosynthetic capacity, not direct evidence of metabolite production or ecological adaptation [27,28].

### 4.1. Diaporthe Represents a Particularly Rich Reservoir of Specialized-Metabolite Biosynthesis

The large number of BGCs identified in the four genomes reinforces the emerging view of *Diaporthe* as a metabolically prolific fungal genus. A review of experimentally reported metabolites identified polyketides as the largest compound group, alongside terpenoids, steroids, lactones, alkaloids and other classes with diverse reported bioactivities [29]. This chemical predominance of polyketides is consistent with our genomic observations, in which T1PKS-associated BGCs constituted the largest biosynthetic category in every isolate. The extensive PKS repertoire is also consistent with the structural diversity of polyketide-derived metabolites experimentally reported from *Diaporthe*, including pyrones, isocoumarins, chromones, quinones, xanthones, and dibenzopyrones [30]. Together, these genomic and chemical observations further support the capacity of *Diaporthe* to generate diverse polyketide scaffolds with a broad range of biological activities. The scale of the BGC repertoires observed here is also noteworthy. The four isolates encoded 112–129 BGCs each, indicating considerably greater biosynthetic potential than can normally be inferred from metabolite profiles obtained under a single cultivation condition. A recent genome-mining study of *D. kyushuensis* ZMU-48-1 identified 98 BGCs, 60% of which lacked similarity to known clusters, while an OSMAC approach yielded 18 metabolites, including two novel pyrrole derivatives, some of which showed antifungal activity [13]. More generally, cultivation changes, co-culture and epigenetic or molecular interventions can reveal fungal pathways that are weakly expressed under routine conditions [4,31,32]. Consequently, the large BGC repertoire identified here should be viewed not simply as a catalog of predicted pathways but as a resource for targeted natural-product discovery.

### 4.2. Biosynthetic Diversity Is Much Greater than Overall Proteomic Diversity

The much stronger conservation of orthologous protein clusters than of GCF membership indicates that related *Diaporthe* genomes may maintain a common functional framework while differing in specialized-metabolite potential. Such variation could arise through differential retention, loss, duplication or remodeling of biosynthetic loci; the present four-genome comparison, however, cannot establish the relative contributions of these processes or of host-associated selection. In particular, *D*. cf. *heveae* LGMF1633 and *D. vochysiae* LGMF1583, although isolated from *Vochysia divergens*, occupy differentiated phylogenetic positions and possess distinct predicted biosynthetic repertoires. Their comparison cautions against attributing biosynthetic similarity or divergence to host associations without accounting for evolutionary relatedness. Functional annotations also suggest that biosynthetic diversification may extend beyond core synthases: cytochrome P450s, oxidoreductases, methyltransferases and other tailoring enzymes can alter scaffold chemistry [33,34], while transcriptional regulators influence pathway activation [35,36]. Nevertheless, the abundance of these protein families across whole proteomes does not establish their participation in particular BGCs. Gene-level characterization and expression studies are needed to connect these capacities to specific metabolites.

### 4.3. Core GCFs Indicate a Shared Biosynthetic Foundation

The identification of core GCFs associated with squalestatin S1 (GCF84), choline (GCF109), scytalone/T3HN (GCF197), cercosporin (GCF214), and nectriapyrone (GCF241) indicates the presence of shared predicted biosynthetic systems across the four *Diaporthe* isolates. These GCFs represent different biosynthetic classes, including terpene-, T1PKS-, and NRPS-like-associated pathways, suggesting that the shared biosynthetic repertoire encompasses multiple specialized-metabolite biosynthetic mechanisms. However, their distribution across all four isolates indicates conservation at the GCF-membership level rather than complete conservation of gene content, cluster organization, or metabolic products. Comparative examination of the predicted BGCs revealed distinct biosynthetic architectures among these core families. The squalestatin S1-associated GCF84 contained genes encoding a squalene synthase with an SQS_PSY domain and DnaJ-family proteins, whereas the cercosporin-associated GCF214 contained a CTB1-like polyketide synthase together with predicted O-methyltransferase-, laccase-, and transporter-associated components. Similarly, the nectriapyrone-associated GCF241 comprised multidomain type I PKS-associated BGCs with methyltransferase-related proteins. These features indicate the presence of shared predicted core biosynthetic machinery accompanied by accessory components potentially involved in biosynthetic modification or metabolite transport. The choline-associated GCF109 and scytalone/T3HN-associated GCF197 further extend the shared repertoire to NRPS-like and additional polyketide-associated systems. The predicted associations with characterized pathways provide useful references for interpreting the biosynthetic potential of these GCFs. Squalestatin-associated pathways are linked to compounds with reported squalene synthase inhibitory and antifungal activities [37,38], while nectriapyrone-associated biosynthetic signatures have previously been identified through genome mining of *Diaporthe* [31]. The experimental identification of nectriapyrone in *D. eres* [39] and alternariol-related metabolites in other *Diaporthe* isolates [40,41] further demonstrates that related biosynthetic systems can occur within the genus. Nevertheless, these observations do not establish production of the corresponding metabolites by the four isolates investigated here. Likewise, predicted associations with cercosporin, alternariol, PR-toxin, and patulin should be interpreted as genomic similarities rather than evidence of toxin biosynthesis or biological activity. Overall, the core GCFs provide a genomic framework for investigating shared biosynthetic potential among the four isolates. Comparative characterization of their core biosynthetic enzymes, tailoring-enzyme complements, and cluster organization, followed by gene-expression analysis, targeted metabolomics, and functional validation, will be necessary to establish their biosynthetic products and determine whether related GCFs encode identical or divergent metabolic pathways.

### 4.4. Isolate-Specific BGCs Reveal Chemical Differentiation

The occurrence of isolate-specific BGC signatures indicates substantial differentiation in the predicted specialized-metabolite repertoires of the four *Diaporthe* isolates. These differences suggest that, despite sharing a substantial proportion of their orthologous protein clusters, the isolates possess distinct combinations of biosynthetic loci potentially contributing to metabolic diversification. Previous investigations have demonstrated considerable variation in the specialized metabolites isolated from different *Diaporthe* species [42,43,44], providing a broader experimental context for the genomic diversity observed in the present study. The isolate-specific pathway-associated predictions encompassed multiple biosynthetic classes, including terpene-, PKS-, NRPS-, and hybrid-associated systems. In particular, the α-acorenol- and phyllostictine-associated BGCs identified in *D. inconspicua*, the chaetolivacine, chrysogine-, patulin-, and AKML-associated BGCs in *D. vochysiae*, and the enniatin-associated BGC in *Diaporthe* sp. HANT25 illustrate differences in predicted biosynthetic capacity among the isolates. The enniatin-associated locus in HANT25 represents a candidate for investigating NRPS-mediated peptide biosynthesis. Although related cyclic peptides have previously been associated with biological activities [45], the genomic similarity observed here does not confirm production of these metabolites. These isolate-specific BGCs provide candidates for further investigation of biosynthetic diversification within *Diaporthe*. In particular, differences in core biosynthetic enzymes, domain organization, tailoring-enzyme composition, and associated regulatory components may influence the chemical structures potentially generated by these loci. Prioritizing clusters with distinctive biosynthetic architectures or limited similarity to characterized reference pathways, followed by transcriptomic profiling, metabolomic analysis, and targeted functional characterization, may help identify previously uncharacterized biosynthetic products. However, the present computational analysis establishes predicted biosynthetic potential rather than experimentally demonstrated metabolite production.

### 4.5. Uncharacterized GCFs Highlight Unexplored Biosynthetic Potential

A major component of the biosynthetic repertoire consisted of GCFs without significant correspondence to characterized MIBiG pathways. Many of these families nevertheless contained recognizable PKS, NRPS, NRPS-like, terpene, or hybrid biosynthetic machinery. Although the absence of a characterized match cannot itself demonstrate chemical novelty, these GCFs represent an important reservoir of chemically unresolved biosynthetic potential. The significance of such uncharacterized pathways is supported by genome-guided studies of *Diaporthe*. In *D. kyushuensis*, genome mining revealed numerous uncharacterized BGCs, while subsequent chemical investigation under altered cultivation conditions uncovered diverse metabolites, including previously undescribed compounds [13]. Likewise, metabolomic investigation of the medicinal plant endophyte *D. caliensis* revealed extensive polyketide-lactone diversity and emphasized the importance of coupling metabolomic predictions with compound isolation and structural characterization [46]. The isolate-restricted uncharacterized GCFs are particularly relevant for future investigation because their restricted distribution may reflect isolate-level biosynthetic diversification. Candidate clusters could be prioritized according to biosynthetic architecture, unusual PKS or NRPS domain organization, hybrid composition, and the presence of tailoring enzymes, pathway-specific regulators, and transporters. Such prioritization would provide a rational framework for linking genome mining to experimental natural-product discovery.

### 4.6. Biotechnological Relevance and Future Characterization

The biosynthetic diversity identified in these *Diaporthe* genomes is relevant to both pharmaceutical and agricultural natural-product discovery. Fungal endophytes are established sources of metabolites with antimicrobial, cytotoxic, anti-inflammatory, antioxidant, antiviral, and enzyme-inhibitory activities, and medicinal plant-associated endophytes represent particularly attractive targets for bioprospecting [31]. Experimental studies have demonstrated comparable bioactivity among *Diaporthe* metabolites, including antibacterial, antifungal, antioxidant, cytotoxic, and other activities [42,47,48]. The identified BGCs may also have relevance to agricultural applications. Endophytic *Diaporthe* species have been proposed as sources of natural products with potential applications in biological control and crop protection [49]. Genome mining combined with OSMAC cultivation of *D. kyushuensis*, for example, identified metabolites active against the plant pathogens Bipolaris sorokiniana and *Botryosphaeria dothidea* [13]. These observations support targeted screening of the present isolates for antimicrobial and phytopathogen-inhibitory compounds, while also emphasizing the need to assess phytotoxicity and effects on non-target organisms before agricultural application.

An important limitation of the present analysis is that BGC prediction establishes biosynthetic potential rather than metabolite production. The presence of a predicted BGC does not establish pathway expression, pathway completeness, production of a specific metabolite, or biological activity. Likewise, similarity to a characterized MIBiG cluster does not necessarily imply that the same compound is produced, because differences in gene content and pathway organization may alter biosynthetic outputs. Gene loss or duplication, divergence of tailoring enzymes, and differences in regulatory components may further contribute to variation among related BGCs. In addition, incomplete genome assemblies or inaccuracies in gene prediction may affect inferred BGC boundaries, gene content, and subsequent GCF assignments. These factors should therefore be considered when interpreting differences in BGC and GCF distributions among the four isolates. Although all four isolates analyzed here are endophytes, the present dataset does not permit identification of endophyte-specific BGCs because saprobic and pathogenic *Diaporthe* isolates were not included in the comparative analysis. Moreover, BGCs restricted to individual isolates in the present dataset should not be interpreted as endophyte-specific, as their distribution may instead reflect species- or strain-level genomic variation. Establishing whether particular BGCs are associated with an endophytic lifestyle will require systematic comparison with phylogenetically representative endophytic, saprobic, and pathogenic *Diaporthe* isolates analyzed using a consistent methodology. Many fungal BGCs remain silent or weakly expressed under standard laboratory conditions. Approaches such as OSMAC cultivation, microbial co-culture, epigenetic modulation, transcription-factor activation, and heterologous expression can facilitate access to such cryptic pathways. OSMAC approaches have already proven effective in *Diaporthe*, while epigenetic manipulation has been widely used to activate silent fungal secondary metabolism [14,50]. Heterologous expression and BGC refactoring provide additional strategies for experimentally accessing otherwise inactive pathways [51]. Accordingly, integration of genome mining with LC-HRMS/MS metabolomics provides a logical next step for characterizing the four isolates. Molecular networking and transcriptomic profiling could facilitate associations between metabolite features and candidate BGCs, whereas purification followed by NMR and high-resolution mass spectrometry would be required for definitive structural characterization. Ultimately, genetic disruption or heterologous expression would provide stronger evidence linking individual metabolites to their corresponding biosynthetic pathways. As emphasized by metabolomic analysis of *D. caliensis*, experimental structural characterization remains essential to avoid overinterpretation of automated metabolite annotations [46].

Candidate BGCs can be prioritized for experimental investigation using complementary genomic criteria. Core GCFs with recognizable pathway architecture provide candidates for determining whether shared biosynthetic capacity results in comparable metabolite profiles, whereas GCFs showing divergence from characterized MIBiG pathways, distinctive tailoring-enzyme complements, or unusual core-biosynthetic architectures may represent promising targets for identifying pathway variants or structurally distinct products. Isolate-restricted and chemically uncharacterized GCFs are particularly relevant for investigating isolate-specific biosynthetic capacity. Integrating these genomic criteria with transcriptomic and metabolomic evidence would provide a rational framework for selecting candidate BGCs for subsequent functional characterization.

Overall, the comparative analysis demonstrates that medicinal plant-associated endophytic *Diaporthe* possess a conserved biosynthetic foundation accompanied by substantial isolate-level diversification. Particularly important are the chemically uncharacterized and isolate-restricted GCFs, which provide candidates for future metabolomic and functional investigations. Integrating comparative genomics with culture-based induction, metabolomics, bioactivity screening, and experimental pathway validation should help determine which of these predicted BGCs encode biologically active or structurally distinct natural products and clarify their potential pharmaceutical, agricultural, and biotechnological relevance.

## 5. Conclusions

Comparative genomic analysis of four medicinal plant-associated endophytic *Diaporthe* isolates revealed a conserved proteomic background coupled with substantial diversification of specialized-metabolite biosynthetic potential. Although 69.3% of orthologous protein clusters were shared across all four isolates, the 481 predicted BGCs were grouped into 348 GCFs, of which only 15 were represented in all four genomes. The predominance of T1PKS, NRPS, NRPS-like, terpene, and hybrid biosynthetic systems, together with the diversity of associated tailoring enzymes, highlights their extensive predicted biosynthetic capacity. Core GCFs and isolate-restricted biosynthetic signatures further indicate both shared and isolate-dependent biosynthetic potential, while the substantial proportion of chemically uncharacterized GCFs suggests opportunities for genome-guided natural-product discovery. Importantly, the identified BGCs and GCFs represent computationally predicted candidate biosynthetic loci rather than experimentally validated pathways, and their expression, metabolite products, and biological activities remain to be established. Future investigations should prioritize candidate BGCs based on their shared or isolate-restricted distribution, divergence from characterized MIBiG pathways, distinctive tailoring-enzyme complements, and unusual core biosynthetic architectures. Targeted gene-expression analysis and LC-MS/MS-based metabolomics, followed by genetic disruption or heterologous expression of selected clusters, will be essential to establish their biosynthetic functions and products.

## Figures and Tables

**Table 1:** Genome characteristics of *Diaporthe* species used in this study.

Species/Isolate	NCBI/JGI id	Host Plant	Genome Size (Mb)	Total Proteins	Scaffolds	N50 (Mb)	GC Content (%)	BGCs
*Diaporthe inconspicua* LGMF1612	Diainc1	*Maytenus ilicifolia*	56.26	16,795	20	5.57	52.31	117
*Diaporthe* cf. *heveae* LGMF1633	DiaLGMF1633	*Vochysia divergens*	52.45	16,033	12	5.01	52.73	123
*Diaporthe vochysiae* LGMF1583	Diavoc1	*Vochysia divergens*	60.11	17,434	10	8.38	51.68	112
*Diaporthe* sp. HANT25	GCA_013435955.1	*Hydnocarpus anthelminthicus*	55.34	16,531	79	2	52.11	129

**Table 2:** Distribution of biosynthetic gene cluster (BGC) classes across the four medicinal plant-associated endophytic *Diaporthe* isolates.

BGC Class	*D. inconspicua *LGMF1612	*D. *cf. *heveae* LGMF1633	*D. vochysiae *LGMF1583	*Diaporthe* sp. HANT25
T1PKS	53	57	54	58
T3PKS	2	2	1	2
NRPS	22	17	25	24
NRPS-like	19	22	18	20
Terpene	37	40	24	34
Indole	9	7	8	6
Fungal RiPP	2	2	0	2
Terpene precursor	2	2	2	2
β-Lactone	1	2	1	1
Isocyanide	1	1	1	1
Nitropropanoic acid	1	1	0	1
NRP-metallophore	0	1	1	1
NI-siderophore	1	1	1	1
Other	1	2	1	1
Cytokinin	0	1	0	0
Total BGC regions	117	123	112	129

Note: BGC-class counts are not mutually exclusive because individual antiSMASH regions may contain more than one biosynthetic class (hybrid/multiclass BGCs). Consequently, the sum of the individual class counts may exceed the total number of predicted BGC regions.

## Data Availability

The original contributions presented in this study are included in the article/Supplementary Material. Further inquiries can be directed to the corresponding author.

## Supplementary Materials

The following supporting information can be downloaded at https://www.mdpi.com/article/10.3390/jof12100784/s1; Table S1: Gene annotation of selected species; Table S2: Functional annotation of *Diaporthe* species; Table S3: Orthogroups in four *Diaporthe* species; Table S4: Biosynthetic gene clusters identified in *Diaporthe* species; Table S5: Gene cluster family (GCF) distribution, MIBiG pathway associations, and GCF-level characterization across four endophytic Diaporthe isolates. Table S6: antiSMASH KnownClusterBlast evidence supporting MIBiG-associated pathway assignments of five core GCFs represented across all four Diaporthe isolates.

## References

[1] Liao C., Doilom M., Jeewon R., Hyde K.D., Manawasinghe I.S., Chethana K.W.T., Balasuriya A., Thakshila S.A.D., Luo M., Mapook A. (2025). Challenges and Update on Fungal Endophytes: Classification, Definition, Diversity, Ecology, Evolution and Functions. Fungal Divers..

[2] Sharma M., Bharti S., Goswami A., Mallubhotla S. (2023). Diversity, Antimicrobial, Antioxidant, and Anticancer Activity of Culturable Fungal Endophyte Communities in *Cordia dichotoma*. Molecules.

[3] Gupta A., Meshram V., Gupta M., Goyal S., Qureshi K.A., Jaremko M., Shukla K.K. (2023). Fungal Endophytes: Microfactories of Novel Bioactive Compounds with Therapeutic Interventions; A Comprehensive Review on the Biotechnological Developments in the Field of Fungal Endophytic Biology over the Last Decade. Biomolecules.

[4] Zakariyah R.F., Ajijolakewu K.A., Ayodele A.J., Folami-A B.I., Samuel E.P., Otuoze S.O., Abdulrauf L.B., Ahmed R.N. (2024). Progress in Endophytic Fungi Secondary Metabolites: Biosynthetic Gene Cluster Reactivation and Advances in Metabolomics. Bull. Natl. Res. Cent..

[5] Yan D.-H., Song X., Li H., Luo T., Dou G., Strobel G. (2018). Antifungal Activities of Volatile Secondary Metabolites of Four *Diaporthe* Strains Isolated from *Catharanthus roseus*. J. Fungi.

[6] Mayrhofer B.F., Iantas J., Noriler S.A., Ponomareva L.V., Thorson J.S., Rohr J., Shaaban K.A., Glienke C. (2024). Highly Diverse Endophytic Fungi from Serra Do Amolar-Pantanal (Brazil) Producing Bioactive Secondary Metabolites against Phytopathogens. Front. Microbiol..

[7] Petijová L., Henzelyová J., Kuncová J., Matoušková M., Čellárová E. (2024). In Silico Prediction of Polyketide Biosynthetic Gene Clusters in the Genomes of Hypericum-Borne Endophytic Fungi. BMC Genom..

[8] Minami A., Ugai T., Ozaki T., Oikawa H. (2020). Predicting the Chemical Space of Fungal Polyketides by Phylogeny-Based Bioinformatics Analysis of Polyketide Synthase-Nonribosomal Peptide Synthetase and Its Modification Enzymes. Sci. Rep..

[9] Koczyk G., Pawłowska J., Muszewska A. (2021). Terpenoid Biosynthesis Dominates among Secondary Metabolite Clusters in Mucoromycotina Genomes. J. Fungi.

[10] Brakhage A.A., Schroeckh V. (2011). Fungal Secondary Metabolites—Strategies to Activate Silent Gene Clusters. Fungal Genet. Biol..

[11] Keller N.P. (2019). Fungal Secondary Metabolism: Regulation, Function and Drug Discovery. Nat. Rev. Microbiol..

[12] Rokas A., Mead M.E., Steenwyk J.L., Raja H.A., Oberlies N.H. (2020). Biosynthetic Gene Clusters and the Evolution of Fungal Chemodiversity. Nat. Prod. Rep..

[13] Zheng J., Wang H., Wang X., Zeng S., Yuan S., Yin T. (2025). Genome Mining and OSMAC Strategies Unveil Diverse Secondary Metabolites from the Endophytic Fungus *Diaporthe kyushuensis* ZMU-48-1 with Antifungal Activity. Front. Microbiol..

[14] Li K., Zhang C., Zhang Z., Cheng J., Gao Q., Gao L., Zhao X., Yan W., Wang Y., Ye W. (2026). Telomere-to-Telomere Genomes Reveal That Multiscale Evolution Shapes the Largest Metabolic Arsenal of *Diaporthe* Fungi. Adv. Sci..

[15] Tulsook K., Isarangkul D., Sriubolmas N., Kittakoop P., Wiyakrutta S. (2020). Draft Genome Sequence of *Diaporthe* sp. Strain HANT25, an Endophytic Fungus Producing Mycoepoxydiene. Microbiol. Resour. Announc..

[16] Noriler S.A., Savi D.C., Ponomareva L.V., Rodrigues R., Rohr J., Thorson J.S., Glienke C., Shaaban K.A. (2019). Vochysiamides A and B: Two New Bioactive Carboxamides Produced by the New Species *Diaporthe vochysiae*. Fitoterapia.

[17] Noriler S.A., Savi D.C., Aluizio R., Palácio-Cortes A.M., Possiede Y.M., Glienke C. (2018). Bioprospecting and Structure of Fungal Endophyte Communities Found in the Brazilian Biomes, Pantanal, and Cerrado. Front. Microbiol..

[18] Gomes R.R., Glienke C., Videira S.I.R., Lombard L., Groenewald J.Z., Crous P.W. (2013). *Diaporthe*: A Genus of Endophytic, Saprobic and Plant Pathogenic Fungi. Persoonia.

[19] Zhu Y., Ma L., Xue H., Li Y., Jiang N. (2024). New Species of *Diaporthe* (Diaporthaceae, Diaporthales) from *Bauhinia variegata* in China. MycoKeys.

[20] Tamura K., Stecher G., Kumar S. (2021). MEGA11: Molecular Evolutionary Genetics Analysis Version 11. Mol. Biol. Evol..

[21] Sun J., Lu F., Luo Y., Bie L., Xu L., Wang Y. (2023). OrthoVenn3: An Integrated Platform for Exploring and Visualizing Orthologous Data across Genomes. Nucleic Acids Res..

[22] Cantalapiedra C.P., Hernández-Plaza A., Letunic I., Bork P., Huerta-Cepas J. (2021). EggNOG-Mapper v2: Functional Annotation, Orthology Assignments, and Domain Prediction at the Metagenomic Scale. Mol. Biol. Evol..

[23] Blin K., Shaw S., Vader L., Szenei J., Reitz Z.L., Augustijn H.E., Cediel-Becerra J.D.D., de Crécy-Lagard V., Koetsier R.A., Williams S.E. (2025). AntiSMASH 8.0: Extended Gene Cluster Detection Capabilities and Analyses of Chemistry, Enzymology, and Regulation. Nucleic Acids Res..

[24] Zdouc M.M., Blin K., Louwen N.L.L., Navarro J., Loureiro C., Bader C.D., Bailey C.B., Barra L., Booth T.J., Bozhüyük K.A.J. (2025). MIBiG 4.0: Advancing Biosynthetic Gene Cluster Curation through Global Collaboration. Nucleic Acids Res..

[25] Kautsar S.A., van der Hooft J.J.J., de Ridder D., Medema M.H. (2021). BiG-SLiCE: A Highly Scalable Tool Maps the Diversity of 1.2 Million Biosynthetic Gene Clusters. Gigascience.

[26] Gilchrist C.L.M., Chooi Y.-H. (2021). Clinker & Clustermap.Js: Automatic Generation of Gene Cluster Comparison Figures. Bioinformatics.

[27] Takahashi H., Umemura M., Ninomiya A., Kusuya Y., Shimizu M., Urayama S., Watanabe A., Kamei K., Yaguchi T., Hagiwara D. (2021). Interspecies Genomic Variation and Transcriptional Activeness of Secondary Metabolism-Related Genes in Aspergillus Section Fumigati. Front. Fungal Biol..

[28] Steenwyk J.L., Mead M.E., Knowles S.L., Raja H.A., Roberts C.D., Bader O., Houbraken J., Goldman G.H., Oberlies N.H., Rokas A. (2020). Variation Among Biosynthetic Gene Clusters, Secondary Metabolite Profiles, and Cards of Virulence Across *Aspergillus* Species. Genetics.

[29] Xu T.-C., Lu Y.-H., Wang J.-F., Song Z.-Q., Hou Y.-G., Liu S.-S., Liu C.-S., Wu S.-H. (2021). Bioactive Secondary Metabolites of the Genus *Diaporthe* and Anamorph Phomopsis from Terrestrial and Marine Habitats and Endophytes: 2010–2019. Microorganisms.

[30] Wei W., Khan B., Dai Q., Lin J., Kang L., Rajput N.A., Yan W., Liu G. (2023). Potential of Secondary Metabolites of *Diaporthe* Species Associated with Terrestrial and Marine Origins. J. Fungi.

[31] Hashem A.H., Attia M.S., Kandil E.K., Fawzi M.M., Abdelrahman A.S., Khader M.S., Khodaira M.A., Emam A.E., Goma M.A., Abdelaziz A.M. (2023). Bioactive Compounds and Biomedical Applications of Endophytic Fungi: A Recent Review. Microb. Cell Fact..

[32] Verma A., Tiwari H., Singh S., Gupta P., Rai N., Kumar Singh S., Singh B.P., Rao S., Gautam V. (2023). Epigenetic Manipulation for Secondary Metabolite Activation in Endophytic Fungi: Current Progress and Future Directions. Mycology.

[33] Chiang C.-Y., Ohashi M., Tang Y. (2023). Deciphering Chemical Logic of Fungal Natural Product Biosynthesis through Heterologous Expression and Genome Mining. Nat. Prod. Rep..

[34] Zhang L., Fasoyin O.E., Molnár I., Xu Y. (2020). Secondary Metabolites from Hypocrealean Entomopathogenic Fungi: Novel Bioactive Compounds. Nat. Prod. Rep..

[35] Wang W., Yu Y., Keller N.P., Wang P. (2021). Presence, Mode of Action, and Application of Pathway Specific Transcription Factors in Aspergillus Biosynthetic Gene Clusters. Int. J. Mol. Sci..

[36] Zhgun A.A. (2023). Fungal BGCs for Production of Secondary Metabolites: Main Types, Central Roles in Strain Improvement, and Regulation According to the Piano Principle. Int. J. Mol. Sci..

[37] Dawson M.J., Farthing J.E., Marshall P.S., Middleton R.F., O’Neill M.J., Shuttleworth A., Stylli C., Tait R.M., Taylor P.M., Wildman H.G. (1992). The squalestatins, novel inhibitors of squalene synthase produced by a species of *Phoma*. I. Taxonomy, fermentation, isolation, physico-chemical properties and biological activity. J. Antibiot..

[38] Baxter A., Fitzgerald B.J., Hutson J.L., McCarthy A.D., Motteram J.M., Ross B.C., Sapra M., Snowden M.A., Watson N.S., Williams R.J. (1992). Squalestatin 1, a Potent Inhibitor of Squalene Synthase, Which Lowers Serum Cholesterol in Vivo. J. Biol. Chem..

[39] Reveglia P., Pacetti A., Masi M., Cimmino A., Carella G., Marchi G., Mugnai L., Evidente A. (2021). Phytotoxic Metabolites Produced by *Diaporthe eres* Involved in Cane Blight of Grapevine in Italy. Nat. Prod. Res..

[40] Guo L., Niu S., Chen S., Liu L. (2020). Diaporone A, a New Antibacterial Secondary Metabolite from the Plant Endophytic Fungus *Diaporthe* sp. J. Antibiot..

[41] Mountessou B.Y.G., Anoumedem É.G.M., Kemkuignou B.M., Marin-Felix Y., Surup F., Stadler M., Kouam S.F. (2023). Secondary Metabolites of *Diaporthe cameroonensis*, Isolated from the Cameroonian Medicinal Plant *Trema guineensis*. Beilstein J. Org. Chem..

[42] Gu H., Zhang S., Liu L., Yang Z., Zhao F., Tian Y. (2022). Antimicrobial Potential of Endophytic Fungi from *Artemisia argyi* and Bioactive Metabolites from *Diaporthe* sp. AC1. Front. Microbiol..

[43] Zhai G., Chen S., Shen H., Guo H., Jiang M., Liu L. (2022). Bioactive Monoterpenes and Polyketides from the Ascidian-Derived Fungus *Diaporthe* sp. SYSU-MS4722. Mar. Drugs.

[44] Chen Y., Wang J., Zhao C.-L., Yang X.-F., Ye J., Zhang L.-P., Li Y.-X., Liu F.-W., Zhang L., Song B. (2026). Two New Monoterpene Glycosides from the Endophytic Fungus *Diaporthe nobilis* and Their Cytotoxic Effects. Nat. Prod. Res..

[45] Yu Z., Nong X., Wei S., Wu G., Qin Q., Tan H. (2025). Phomopamide A, a Cyclic Pentadepsipeptide with α-Glucosidase Inhibition Activity from the Endophytic Fungus *Diaporthe* sp. Nat. Prod. Res..

[46] Charria-Girón E., Marin-Felix Y., Beutling U., Franke R., Brönstrup M., Vasco-Palacios A.M., Caicedo N.H., Surup F. (2023). Metabolomics Insights into the Polyketide-Lactones Produced by *Diaporthe* caliensis sp. Nov., an Endophyte of the Medicinal Plant *Otoba gracilipes*. Microbiol. Spectr..

[47] Santra H.K., Roy A., Dutta R., Banerjee D. (2026). Secondary Metabolites from *Diaporthe eres* SK3, an Endophytic Fungus of *Sellaginella kraussiana* Sw. Collected from the Northeast Indian Hills, Exhibits Potential in Vitro Bioactivities. Curr. Microbiol..

[48] Magagula M., Motaung T.E., Mbita Z., Dithebe K. (2025). Investigating the Antimicrobial and Anticancer Potential of Culturable Fungal Endophytes Isolated from the Stems of *Kirkia acuminata* Oliv. BMC Microbiol..

[49] Hilário S., Gonçalves M.F.M. (2022). Endophytic *Diaporthe* as Promising Leads for the Development of Biopesticides and Biofertilizers for a Sustainable Agriculture. Microorganisms.

[50] Xue M., Hou X., Fu J., Zhang J., Wang J., Zhao Z., Xu D., Lai D., Zhou L. (2023). Recent Advances in Search of Bioactive Secondary Metabolites from Fungi Triggered by Chemical Epigenetic Modifiers. J. Fungi.

[51] Li L., Maclntyre L.W., Brady S.F. (2021). Refactoring Biosynthetic Gene Clusters for Heterologous Production of Microbial Natural Products. Curr. Opin. Biotechnol..

